# Daily positive and negative affect during the COVID-19 pandemic

**DOI:** 10.3389/fpsyg.2023.1239123

**Published:** 2024-01-08

**Authors:** Zorana Ivcevic, Shuting Shen, Shengjie Lin, David Cheng, Ryan Probasco, Ben Silbermann, Feng Zhang, Xihong Lin, Marc Brackett

**Affiliations:** ^1^Yale Center for Emotional Intelligence, New Haven, CT, United States; ^2^Department of Biostatistics, Harvard T.H. Chan School of Public Health, Boston, MA, United States; ^3^The How We Feel Project, San Francisco, CA, United States; ^4^Broad Institute of MIT and Harvard, Cambridge, MA, United States; ^5^Department of Biological Engineering, McGovern Institute for Brain Research and Department of Brain and Cognitive Sciences, Massachusetts Institute of Technology, Cambridge, MA, United States; ^6^Howard Hughes Medical Institute (HHMI), Chevy Chase, MD, United States; ^7^Department of Statistics, Harvard University, Cambridge, MA, United States

**Keywords:** positive affect, negative affect, COVID-19 pandemic, demographic differences, stressors, protective behavior

## Abstract

The COVID-19 pandemic influenced emotional experiences globally. We examined daily positive and negative affect between May/June 2020 and February 2021 (N = 151,049; 3,509,982 observations) using a convenience sample from a national mobile application-based survey that asked for daily affect reports. Four questions were examined: (1) How did people in the United States feel from May/June 2020 to February 2021?; (2) What demographic variables are related to positive and negative affect?; (3) What is the relationship between experienced stressors and daily affect?; and (4) What is the relationship between daily affect and preventive behavior? Positive affect increased, and negative decreased over time. Demographic differences mirrored those from before the pandemic (e.g., younger participants reported more negative and less positive affect). Stressors such as feeling unwell, experiencing COVID-19 symptoms, exposure to COVID-19, and lack of sleep were associated with less positive and more negative affect. Exercising protective behaviors predicted future affect, and affect also predicted future protective behaviors (e.g., less protective behavior when happy but more when grateful and thoughtful). The implications for public health communication were discussed.

## Introduction

Experiences of positive and negative affect matter. Affect is an umbrella term that describes feelings (general physical and psychological experiences), moods (relatively longer-lasting, less intense, and somewhat diffused experiences), and emotions (short-term experiences such as surprise or anger that are generally induced by our appraisals of internal or external events; Lazarus and Folkman, 1984; Scherer, 2005; Schwartz and Clore, 2007; Folkman, 2013). Experience of affect varies on the dimensions of valence (from negative to positive) and activation (from low to high activation; Posner et al., 2005). Importantly, affect influences cognitive processes, including memory and decision-making, directing attention toward different information (Forgas and Koch, 2013), and it is key to well-being (Kashdan et al., 2008). The daily experience of positive affect predicts biological markers of cardiovascular health (Tsenkova et al., 2008) and the use of effective emotion regulation that helps cardiovascular recovery after stressful events (Tugade and Fredrickson, 2004). On the other hand, feelings of exhaustion and stress are symptoms of burnout, and routine experience of overall negative affect is a source of vulnerability to mental health disorders such as anxiety, depression, and substance use (Bienvenu et al., 2004). Thus, understanding the course of people’s positive and negative affect during the COVID-19 pandemic and their variations across social groups is relevant to a broad range of behaviors and outcomes.

Understanding daily positive and negative affect can provide insights into possible affective risk factors for mental and physical health problems and the interventions needed in the pandemic aftermath. The COVID-19 pandemic provides a way to examine affect during the experience of global major life stressors. Major life stressors tend to increase negative and lower positive affect, but the majority of those who have experienced extremely bad life circumstances report more positive than negative affect (Diener et al., 2018). When public health policies impose social distancing, many everyday behaviors that enhance positive affect and reduce negative affect are disrupted, which could affect the balance of positive and negative affect. Most pandemic research has focused on mental health outcomes (meta-analyses: Luo et al., 2020; Ren et al., 2020; Wu et al., 2021). The pandemic time pattern of discrete positive and negative affect and how they relate to demographic variables, exposure to pandemic-related stressors, and preventive behaviors remain unclear. To examine affect during the COVID-19 pandemic, we have used daily reports of six positive and six negative feelings collected through the How We Feel smartphone app (HWF; https://www.howwefeel.org), developed for collecting COVID-19-relevant information (Allen et al., 2020). This is a convenience sample, but it is sufficiently large to provide information from the most serious part of the pandemic, namely, from the stay-at-home orders to just before the first vaccines became widely available.

Unexpected events pose a practical challenge to research. Because they are not predictable, it is not possible to plan a research design in which pre-event (in this case, pre-pandemic) assessments could be compared to post-event ones. Occasionally, data are collected for unrelated studies before an event, and it becomes possible to compare them to post-event assessments. For example, Farach et al. (2008) administered a measure of generalized anxiety as a screening measure in a sample of university students in New York City on September 10, 2001. After the terrorist attacks on September 11, the screening data could be used as a pre-test in a longitudinal study of the impacts of a major life stressor. However, such opportunities for pre- and post-designs are rare. The present study includes data from nine months during the COVID-19 pandemic but does not include a pre-pandemic assessment. Thus, the study is relevant to the understanding of the pandemic time affect and the changes throughout this period of the pandemic, but the design does not allow us to speak directly about the pandemic impact (e.g., to what extent the pandemic increased negative affect). The importance of this study is in its ability to inform about specific affect during the height of the pandemic.

### Affect during the COVID-19 pandemic

Research on affect during the COVID-19 pandemic has shown an initial low and subsequent improvement in affect. Representative samples from the U.S. showed increased psychological distress and depression at the pandemic onset (Holman et al., 2020; McGinty et al., 2020). A study that was able to compare January 2020 (pre-pandemic) assessments to March and April 2020 found no change in overall loneliness (Luchetti et al., 2020). In examining specific aspects of loneliness, there was less availability of social connections at the start of the pandemic but a slight decrease in feeling isolated. Other studies found an increase in loneliness from April to May but a leveling off in June 2020 (Killgore et al., 2020).

On a global scale, the Gallup World Happiness Report examined ratings of the extent to which people in 156 countries experienced positive (laughter, enjoyment) and negative affect (worry, anger, sadness) in the previous day (Helliwell et al., 2021) and found increases of worry and sadness after March 2020 compared to 2017–19. The Gallup National Health and Well-Being Index (Witters and Agrawal, 2021), based on weekly assessments of positive and negative affect during the course of the pandemic, pointed to large spikes in ratings of stress and worry in March 2020 (increases more than four times larger than during the 2008 financial crisis), followed by a decline and return to pre-pandemic levels in the first half of 2021. Positive affect similarly showed a large drop in March 2020, followed by an increase, although not reaching pre-pandemic levels. Based on this research, we hypothesized that between May 2020 and February 2021, positive affect would have increased, while negative affect would have decreased. While we were not able to make a comparison with pre-pandemic affect in our study, we predicted that by February 2021, positive affect would have been rated above the neutral point.

### Demographic differences in affect

In the pre-pandemic period, differences in affect were identified for age, gender, and race/ethnicity. Older people experience more positive and less negative affect due to a greater ability to regulate feelings and select situations that align with personal goals (Urry and Gross, 2010; Sims et al., 2015; Burr et al., 2021). Women experience more negative and less positive affect than men (Terracciano et al., 2003; Simon and Nath, 2004). With respect to specific feelings, women tend to report feeling sad, anxious, and angry more often than men, while men tend to report being happy, excited, and calm more than women (Ross and Van Willigen, 1996; Stevenson and Wolfers, 2009; Simon and Lively, 2010). Much less research exists on differences in affective experiences among race and ethnicity groups. The available research shows that African Americans report more positive and less negative affect in retrospective and in-the-moment assessments than White participants (Carstensen et al., 2000; Consedine and Magai, 2002) and are more likely to endorse positive affect in the presence of negative affect (Lankarani and Assari, 2017).

COVID-19 pandemic research shows similar demographic differences. Age correlates with more positive and less negative affect, even after controlling for infection risk, complications, and trait emotional stability (Carstensen et al., 2020). Women tend to report more negative and less positive affect (Carstensen et al., 2020) and greater mental health symptoms (Luo et al., 2020).

Occupational category was found to be important during the COVID-19 pandemic because of the higher infection exposure risk in healthcare and essential workers. Meta-analyses show higher prevalence of affective mental health problems in medical personnel than in the general population (Luo et al., 2020; Wu et al., 2021). However, healthcare and essential workers were less socially isolated and engaged in work that strengthens meaning and purpose (Allan, 2017; Shechter et al., 2020), which might contribute to the higher positive affect compared to non-essential workers.

### Stressors and affect

Bridgland et al. (2021) argued that the COVID-19 pandemic constitutes a traumatic stressor. While the pandemic does not neatly fit the pathogenic event model that attributes traumatic stress reactions to past and primarily direct exposure to life-threatening events, emerging research shows evidence of PTSD-like symptoms as a reaction to COVID-19. Ettman et al. (2022) found that individuals who had more stressors during the pandemic reported more depression symptoms. The present study examines exposure to multiple categories of stressors to test whether greater exposure to pandemic-related stressors is related to higher negative and lower positive affect.

The most common stressors in a national survey study were concerns about COVID-19 exposure (McGinty et al., 2020), and they were associated with experience of distress (Guo et al., 2020; Tanoue et al., 2020). In nationally representative samples in March and April 2020, more depression symptoms were related to prior physical and mental health diagnoses and greater personal exposure to COVID-19 but not to higher community exposure (Holman et al., 2020). During the COVID-19 pandemic, insomnia correlated with mental health symptoms (Shi et al., 2020), distress, and negative affect (Franceschini et al., 2020; Dai et al., 2021). This research aligns with prior studies that showed that sleep deprivation is a stressor associated with lower positive affect and higher anxiety (Talbot et al., 2010). We hypothesize that more negative affect and less positive affect are related to the extent of personal exposure, number of pre-existing health condition stressors, number of experienced physical symptoms, and sleep stressors.

### Affect and preventive behavior

The feelings-as-information model describes affect as data that can inform thinking and action (Clore et al., 1994; Schwartz, 2012). Happiness signals favorable conditions in one’s environment, which leads to lower attention to risks and reducing effort invested in relevant behavior. Whereas happiness is a present-oriented experience (Baumeister et al., 2013), hope and optimism are future-oriented experiences. Optimism is a generalized expectation of positive future outcomes (Carver and Scheier, 1998), and hope includes a positive evaluation of one’s capacity to face goals and having different ways to reach them (Snyder et al., 2002).

By contrast, negative affect signals problems in the environment. Fear and anxiety convey threat and uncertainty, respectively, and thus lower risky behavior (Kaplow et al., 2001) and increased preventive behavior (Hay et al., 2006). Stress, however, is an ambiguous feeling. Although experienced as unpleasant, its effects depend on the appraisal of one’s capacity to cope (Lazarus and Folkman, 1984; Folkman, 2013). If preventive behavior, such as socially distancing, is perceived as reducing infection risk, people will likely engage in those behaviors when stressed.

Krekel et al. (2020) found that the regional life satisfaction assessed by a Gallup World Poll in 2019 predicted less mobility in retail, recreation, transit, and workplaces during the strict lockdown period in 2020 based on mobile phone location data. Self-reported life satisfaction was also related to concurrent adherence to some preventive behaviors (e.g., using sanitizer, washing hands, avoiding crowded areas) but not others (e.g., staying in, avoiding gatherings, masking). However, life satisfaction predicted less preventive behavior one week later.

Based on the feelings-as-information model, we hypothesized that self-reported daily happiness would predict less adherence to protective measures a week later. However, we did not predict this would generalize to other distinct positive affective states such as being grateful or optimistic. In regard to negative affect, we hypothesized that ratings of daily anxiety would predict more adherence to preventive behavior.

In addition to affect predicting protective behavior, we examined whether adherence to protective behavior predicts affect a week later. Pre-pandemic research shows that everyday behaviors predict affective experiences. In particular, social behavior – time spent with family, friends, and romantic partners – is associated with greater happiness (Diener and Seligman, 2002; Robinson and Martin, 2008; Diener et al., 2018), making it likely that restrictions in social interactions during the pandemic reduced happiness. Staying at home during the pandemic predicted greater distress, anxiety and loneliness, and mental health symptoms (Marroquín et al., 2020; Parola et al., 2020; Tull et al., 2020), and wearing masks predicted negative affect, especially in individualist cultures (Lu et al., 2021; Taylor and Asmundson, 2021). Because of this, we hypothesized that staying at home and wearing facial coverings would predict less positive and more negative affect.

### Overview of the present study

This study collected data from users of the How We Feel smartphone app (HWF; https://www.howwefeel.org) to assess affect from the first wave of the pandemic in May 2020 to February 2021, which was the point when the first vaccines were starting to become widely available. Participants received notifications alerting them to complete daily reports of any physical symptoms and six specific positive and negative affects. The study addresses four research questions and tests the following hypotheses:

1. How did people in the United States feel from May/June 2020 to February 2021? We hypothesized that there was an increase in positive and decrease in negative affect.
2. What demographic variables are related to positive and negative affect? Based on existing research, we hypothesized that demographic differences in affect during the pandemic months investigated mirrored pre-pandemic demographic differences in affect.
3. What is the relationship between experienced stressors and daily affect? We hypothesized that there was a linear relationship between exposure to stressors and (higher) negative and (lower) positive affect.
4. What is the relationship between daily affect and preventive behavior? We hypothesized that preventive behavior predicted later affect and that prior affect predicted later predictive behavior.

## Methods

### Participants and procedure

**Figure 1:**
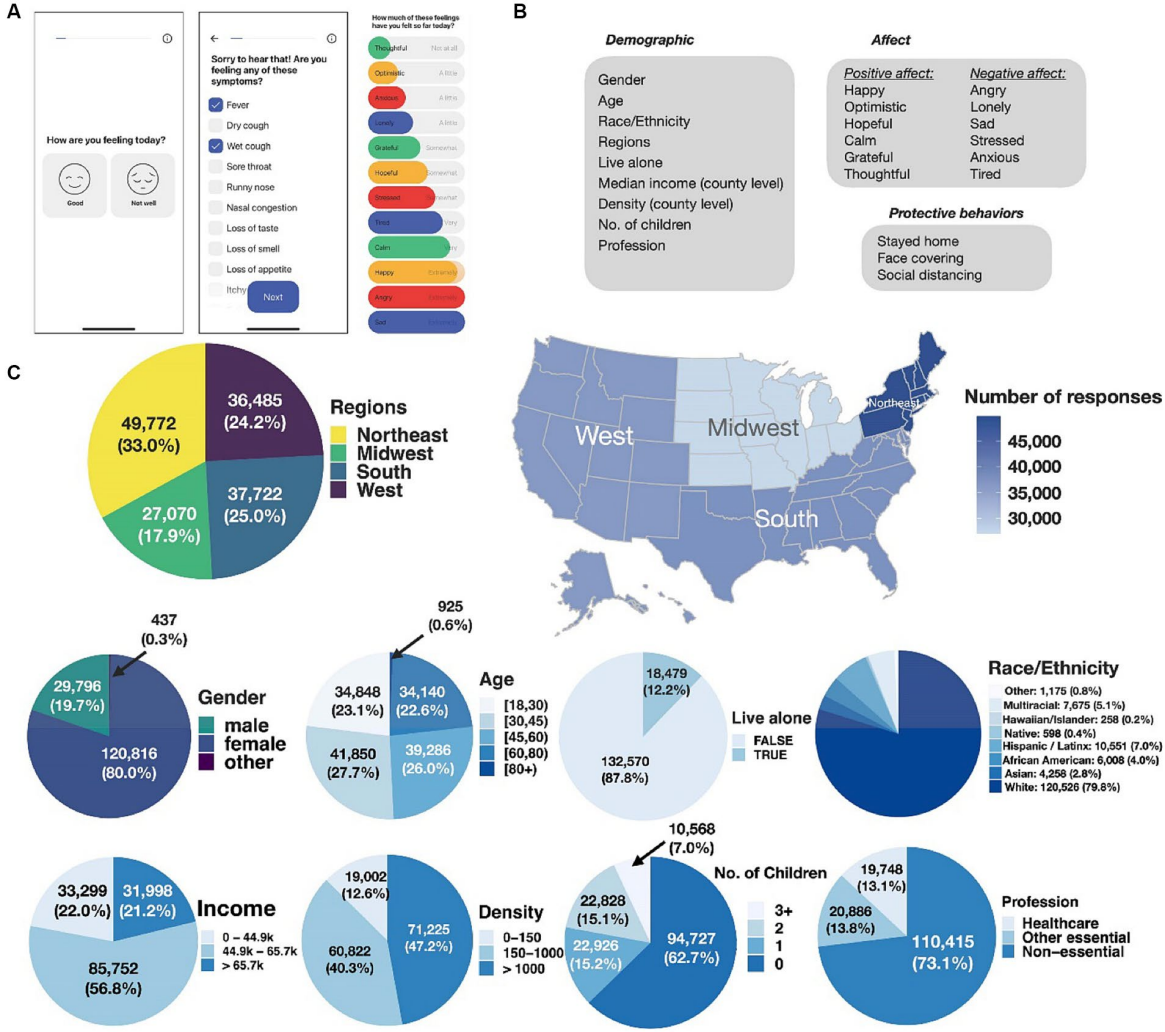
The HWF application and sample note. **(A)** The HWF app: opening screen, COVID-19-related symptoms, affect ratings. **(B)** Demographic, affect, and protective behaviors data collected by the HWF app. **(C)** Distribution of users by geographic region, gender, age, living alone, race/ethnicity, county-level income, population density, number of children in household, and occupational category.

The participants were users of the How We Feel smartphone app (Figure 1), which was developed for collecting COVID-19-relevant information (Allen et al., 2020). Supplementary Table S1 compares the sample demographics with the U.S. Census data when a direct comparison is possible. The sample was largely composed of women (80%) and varied according to age (18–30: 23.1%, 30–45: 27.7%, 45–60: 26%, 60–80: 22.6%, 80+: 0.6%) and geographical region (Northeast: 33%; Midwest: 17.9%; South: 25%; West: 24.2%). Users self-identified as 79.8% White, 7% Hispanic/Latinx, 4% Black/African American, 2.8% Asian/Asian American, 0.4% Native American, 0.2% Hawaiian/Pacific Islander, 5.1% multiracial, and 0.8% other background. Moreover, 13.1% described their occupation as healthcare, 13.8% as other essential jobs, and 73.1% as non-essential.

The HWF app was designed and promoted as a COVID-19 symptom monitoring app, and users were asked to donate their data to researchers to help them learn more about the pandemic. People learned about the app by word of mouth, through online searches for COVID-19 resources, government partnerships (e.g., press release by the governor of Connecticut and associated media coverage), and social media (e.g., advertising on the Pinterest app). Research use of the HWF data was approved (exempt) by the Ethical & Independent Review Services LLP IRB (Study ID 20049–01), the Harvard University Longwood Medical Area Institutional Review Board (IRB) (Protocol no. IRB20-0514), and the Broad Institute of MIT and Harvard IRB (Protocol no. EX-1653). Users were presented a statement, “We’re collecting data from as many people as possible to help health researchers better understand and fight the spread of COVID-19,” and those who consented to donate their data were included in the analyses.

The HWF app assessing daily affect (V.5) was implemented in May 2020. Users were identified only with a device-specific randomly generated number that connected responses at different times. The application was made available only to users 18 or older. Users shared demographic information (gender, age, race/ethnicity, household structure, ZIP code), followed by self-reports of feeling well or not well, physical symptoms, exposure to COVID-19, protective behavior, and daily affect. We excluded people who inconsistently reported gender or had missing location information. Observations without responses to the affect questions were removed. Analyses included data from between May/June 2020 and February 2021, with 151,049 users (3,509,982 observations; average 23.2/participant).

Users were invited to provide daily affect reports. The participants started providing affect reports in May 2020 but could join at any time afterward. Because the participants were app users, retention was dependent on app behavior. According to app industry analyses, most users stop using apps within three months of downloading it (Flynn, 2023). In the present study, 37% of participants who joined in May/June 2020 did not have any reports in July 2020 (see Supplementary Table S2). For users who joined the app in the following months, the drop out from one month to the next was substantially higher, from 60.2% for those who joined in July 2020 to 70.6% for those who joined in January 2021.

Supplementary Figure S1 shows confidence intervals for a logistic regression to assess the association between prior affect and dropping out vs. continuing to use the HWF app (0 = dropping out, 1 = continuing), adjusting for covariates including gender, ethnic groups, age, and months since joining the HWF app. Several significant associations between continuing using the HWF app and prior affect were observed. Participants who continued using the HWF app were less angry and calm and more lonely, sad, anxious, optimistic, hopeful, and thoughtful than those who dropped out. However, continuing to use the app was not systematically related to lower positive and higher negative affect. Among positive affect, continuing to use the app was related to less calm and more optimism, hope, and thoughtfulness. Among negative affect, continuing to use the app was related to less anger and more loneliness and sadness. These results suggest that any potential bias due to prior affect of those who dropped out of participation would be visible only for specific affect variables. Theoretically, we would expect a coupling of positive and negative affect so that changes are similar across positive and negative affect variables. If there is a bias based on who continued to participate, such coupling would likely not be observed.

### Measures

#### Demographics

Users self-reported their gender (options: man, woman, other), age (users entered their age, which was classified into ranges: 18–30, 30–45, 45–60, 60–80, 80+), race/ethnicity (options: American Indian or Alaska Native, Asian, Black or African American, Hispanic/Latinx, Native Hawaiian or Pacific Islander, White, other; those who selected more than one category were reported as multiracial), whether they lived alone (options: yes, no), number of children in the household (options: 0, 1, 2, 3, 4, 5+), and occupational category (coded as healthcare, other essential: construction, critical manufacturing, delivery, essential retail, agriculture and food production, food service, law enforcement/first responders, public works and transportation; and non-essential: retail and malls, personal services, gym or fitness center, child care facility, outdoor attractions and recreation, non-essential office, other).

Location information provided on the app (ZIP codes) was used to extract the county-level median income, density information, and geographic regions from the data by Yu Group (Altieri et al., 2020) at the University of California at Berkeley. Figure 1 shows the distribution of users by geographic region, gender, age, living alone, race/ethnicity, county-level income, population density, number of children in household, and occupational category.

#### Positive and negative affect

Daily affect was assessed for six discrete positive feelings – happy, optimistic, hopeful, calm, grateful, and thoughtful – and six negative feelings – angry, lonely, sad, stressed, anxious, and tired (Figure 1). The scores ranged from 0: “not at all” to 10: “extremely.” Users were asked to rate “How much of these feelings have you felt so far today?” by using slider bars set at 0 by default. Users could indicate the intensity of their feelings by moving the slider to corresponding locations. If a user did not move any slider bars, the affect variables were coded as missing.

#### Stressors

Variables for seven categories of stressors were created:

1. Community exposure. R_t_ value is a key indicator of COVID-19 spread. A value above 1 indicates that infections are on the rise, and a value below 1 indicates a decrease in infections (Amoretti and Lalumera, 2022). We obtained the county-level R_t_ value on the survey date from https://github.com/lin-lab/COVID19-Viz/tree/master/clean_data (Shi et al., 2022). Scores are 0 (outbreak not growing locally) or 1 (outbreak growing locally).
2. Personal exposure. The app asks if the user has been exposed to someone with a confirmed case of COVID-19 and if anyone in the household presents COVID-19 symptoms. Scores range from 0 (no personal exposure) to 2 (both forms of personal exposure selected).
3. Health stressors. The total number of preexisting conditions reported in the app (scores: 0–12).
4. Feeling unwell. When the user opens the app, the first question is, “How are you feeling today?,” with options “good” (scored as 0) and “not well” (scored as 1).
5. Symptom stressors. The total number of common COVID-19 symptoms listed by the CDC reported by the user.^1^ Scores: 0–10.
6. Demographic stressors. The total number of highly affected groups the user belongs to: African American or Hispanic/Latino, age > 65, and living in a high-density area (≥ 150 people per square mile). Scores: 0–3.
7. Sleep stressors. Self-reported hours of sleep the previous night; scores 0 (at least 7–8 h of sleep), 1 (5–6 h of sleep), and 2 (less than 5 h of sleep).

#### Protective behaviors

The users reported if they left home in the past 24 h, and if they did leave home, they were asked if they had taken protective measures, including wearing facial coverings (either a face mask, such as a surgical mask, or another face covering, such as a homemade mask) and social distancing (maintaining at least six feet from others).

#### Statistical modeling

In accordance with the repeated measures nature of the data, we fit a generalized estimating equation (GEE; Liang and Zeger, 1986; Zorn, 2001) linear model to examine the trajectory of affect during the pandemic in the United States. We chose GEE over linear mixed models (LMMs) for the following reasons (Liang and Zeger, 1986; Zorn, 2001; Overall and Tonidandel, 2004; Fitzmaurice et al., 2012): 1. Robustness to misspecification. GEE uses a working correlation structure and is more robust to misspecification of the correlation structure compared to LMMs; 2. No assumption of normality. GEE does not assume that the outcome variable follows a normal distribution, which is an assumption made by LMMs. This flexibility allows GEE to be applied to a wider range of response variables, including those with non-normal distributions; 3. Efficiency with large sample sizes. GEE tends to be more efficient with large sample sizes. It can provide reliable estimates even with a small number of observations within each participant, which might be problematic for LMMs; 4. Computationally less intensive. LMMs involve the estimation of variance–covariance parameters, which can be computationally intensive, especially when dealing with a large number of random effects or when using complex covariance structures.

To address how people in the United States felt, we analyzed each of the 12 affect scores separately using the GEE linear regression. We modeled time effects using dummy variables of months and model covariate effects, including demographics variables, occupation, geographic locations, stressors (e.g., exposure to COVID-19 (reported in app), whether tested for COVID and test results (reported in app), health status), and county-level COVID case rates (obtained from https://github.com/lin-lab/COVID19-Viz/tree/master/clean_data). To alleviate the selection bias of those who completed the affect questions after opening the app, we applied the inverse probability weighting (IPW) adjustments to the GEE regression. IPW is a statistical correction technique that modifies the original regression methods by assigning weights to each observation and conducting the regression based on these weighted observations. Specifically, IPW assigns weights to observations based on their propensity scores, which represent the likelihood of being included in the sample given observed covariates. All observations before drop-off from the survey are included in the logistic regression for fitting the propensity scores. Then, the inverse of the propensity scores is assigned to each observation where users participate in the affect survey as the correction weighting. By appropriately adjusting for these weights in the analysis, unbiased and more precise parameter estimates can be obtained (Seaman and White, 2013).

Next, we performed the IPW GEE linear regression of positive and negative affect ratings on demographics variables, where we adjusted for month dummy variables, sleep duration, whether feeling unwell,^2^ self-reported exposure to COVID-19, testing for COVID and test results, county-level case rates and death rates, and R_t_ values.

The data were analyzed using R version 4.2.2. The R code is included in a Supplementary material.

## Results

The results are presented in four sections addressing each of the research questions.

*RQ1: How did people in the United States feel from May/June 2020 to February 2021?*

**Figure 2:**
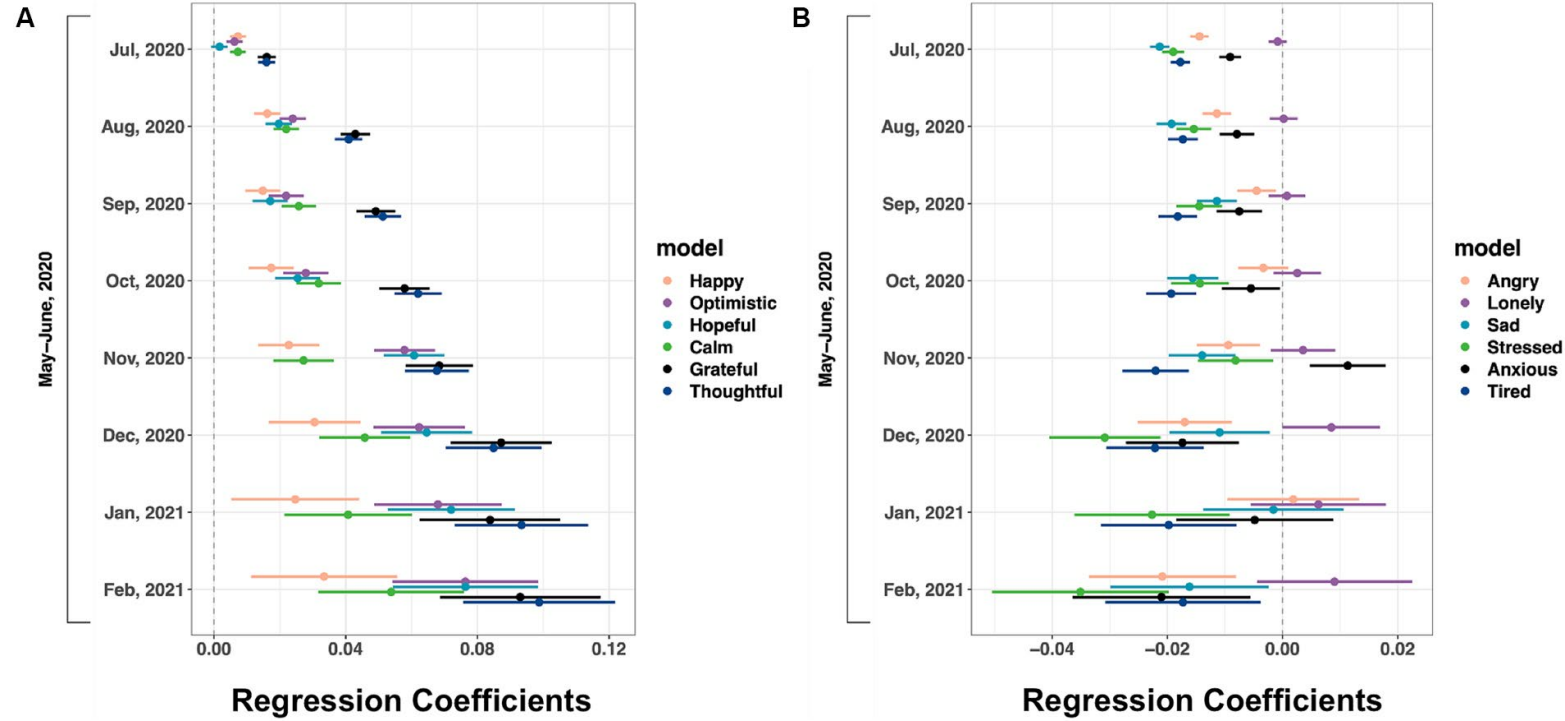
Positive and negative affect during the COVID-19 pandemic compared to May/June 2020. IPW GEE regression of **(A)** positive affect and **(B)** negative affect on pandemic month dummy variables. Covariates: demographics, sleep duration, whether feeling unwell, exposure to COVID-19, testing for COVID and test results, county-level COVID case rates and death rates, and county-level R_t_ values. Shown are 95% confidence intervals (*n* = 3,509,982 responses from 151,049 users). For coefficients, confidence interval values, and *p*-values, see Supplementary Table S3.

Figure 2 presents the mean differences in feelings through the pandemic months compared to May/June 2020 (see Supplementary Table S3 for coefficients, confidence intervals, and *p* values). Positive feelings were higher from August 2020 to February 2021 than in May/June 2020 (February 2021 ranging from thoughtful: coefficient 0.099 to happy: coefficient 0.033). The coefficients got larger over time for positive feelings, indicating increasing positive feelings. Most negative feelings got lower from July 2020 to February 2021 than in May/June 2020, indicating fewer negative feelings over time (February 2021 ranging from sad: coefficient −0.016 to stressed: coefficient −0.035). One exception was loneliness; the coefficients were higher after September 2020 and became significantly higher in December 2020 (coefficient 0.008). There were decreases in anxiety for all months except November 2020 (coefficient: 0.011), possibly associated with landmark events such as the presidential election and delay in the official results.

Supplementary Figure S2 shows that throughout the pandemic, all positive affect was higher than negative affect, indicating an affect balance (ratio of positive and negative affect) greater than 1.

*RQ2: What demographic variables are related to positive and negative affect?*

**Figure 3:**
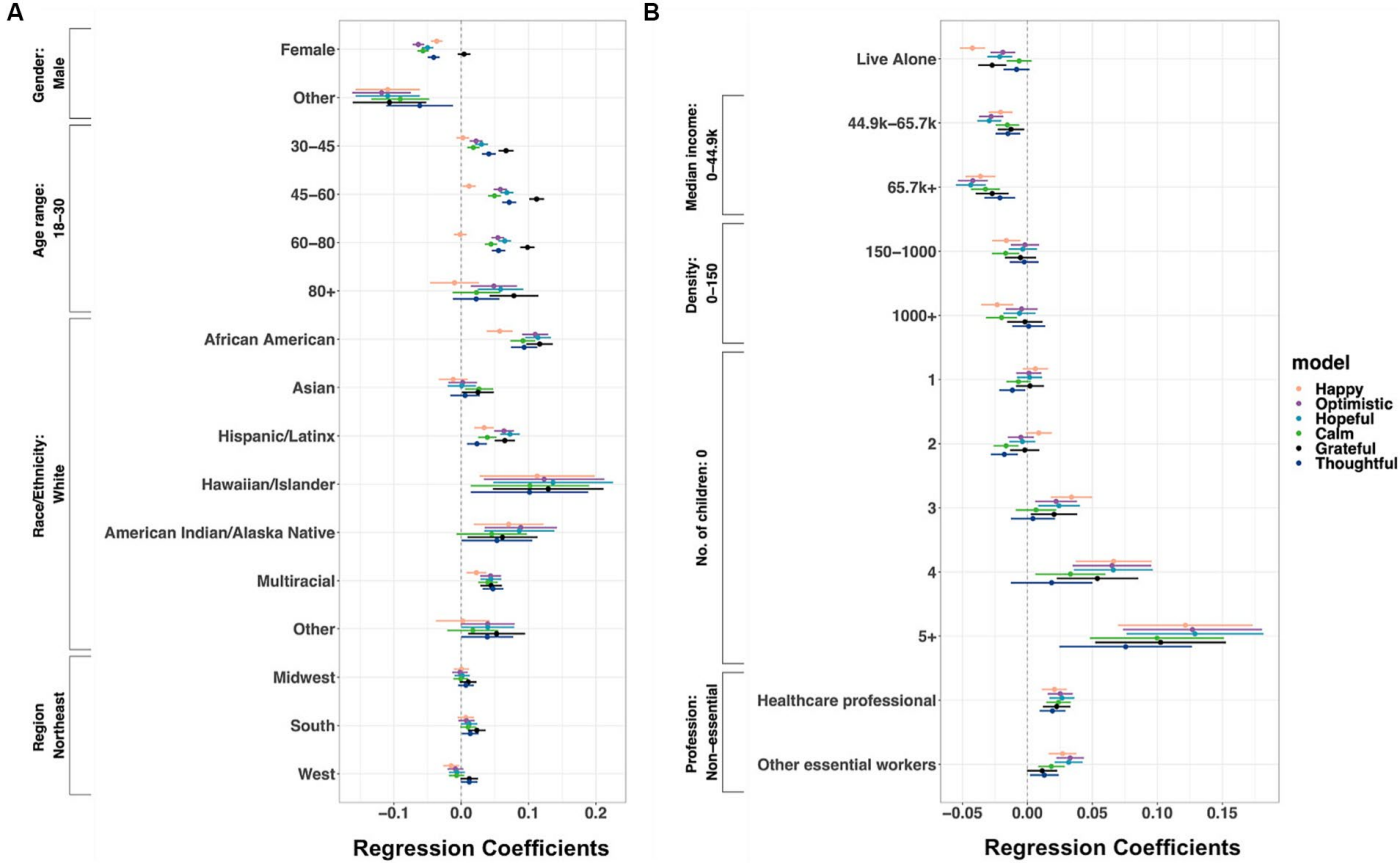
Associations between demographic variables and **(A)** positive and **(B)** negative affect. IPW GEE linear regression of **(A)** positive and **(B)** negative affect on demographics variables. Covariates: month dummy variables, sleep durations, whether feeling unwell, self-reported exposure to COVID-19, testing for COVID and test results, county-level case rates and death rates, and R_t_ values. Shown are estimated coefficients with 95% confidence intervals (*n* = 3,509,982 responses from 151,049 users). For coefficients, confidence interval values, and *p*-values, see Supplementary Table S4.

As shown in Figure 3 (see Supplementary Table S4 for coefficients, confidence intervals, and *p* values), women reported fewer positive feelings than men (ranging from happy: coefficient −0.036 to optimistic: coefficient −0.064). Women were more likely to feel sad (coefficient 0.016), stressed (coefficient 0.015), anxious (coefficient 0.018), and tired (coefficient 0.026) but were less angry (coefficient −0.016) than men.

Older people reported less negative feelings than younger people (compared to 18–30 year olds, ages 30–45: ranging from sad: coefficient −0.019 to tired: coefficient −0.054; ages 45–60: ranging from angry: coefficient −0.024 to tired: coefficient −0.149; ages 60–80: ranging from angry: coefficient −0.050 to tired: coefficient −0.226; ages 80+: ranging from angry: coefficient −0.066 to tired: coefficient −0.256).

Compared to White users, African Americans reported more positive (ranging from happy: coefficient 0.057 to grateful: coefficient 0.116) and less negative feelings (from lonely: coefficient −0.013 to stressed: coefficient −0.064). Similarly, Hispanic/Latinx had more positive (from thoughtful: coefficient 0.023 to hopeful: coefficient 0.072) and less negative feelings (from lonely: coefficient −0.026 to stressed: coefficient −0.043).

Those living alone were lonelier (coefficient 0.073) and sadder (coefficient 0.009) and reported fewer positive feelings (from optimistic: coefficient −0.019 to happy: coefficient −0.042) than those not living alone.

People living in the West reported more negative feelings compared with those in the Northeast, ranging from being stressed: coefficient 0.010 to sad: coefficient 0.022. Those living in counties with a higher median income experienced less positive feelings (compared to income under $44.9 k, $44.9 k-$65.7 k income: ranging from grateful: coefficient −0.013 to hopeful: coefficient −0.029; $65.7 k+: ranging from thoughtful: coefficient −0.021 to hopeful: coefficient −0.044), and those from counties with 1,000+ people per square mile reported more negative feelings compared with those in counties with 0–150 people per square mile (from lonely: coefficient 0.009 to anxious: coefficient 0.021).

Compared to childless participants, those with children reported less anger (from two-child families: coefficient −0.009 to more than five children: coefficient −0.032), loneliness (from two-child families: coefficient −0.013 to more than five children: coefficient −0.020), and sadness (from two-child families: coefficient −0.010 to more than five children: coefficient −0.030) but more stress (from one-child families: coefficient 0.011 to two-child families: coefficient 0.014). Those with more than three children reported more positive feelings than users with no children (three children: ranging from grateful: coefficient 0.020 to happy: coefficient 0.034; four children: from calm: coefficient 0.033 to happy: coefficient 0.066; more than five children: from thoughtful: coefficient 0.076 to hopeful: coefficient 0.129).

Healthcare and other essential workers reported more positive feelings than non-essential workers (healthcare workers, ranging from happy: coefficient 0.021 to hopeful: coefficient 0.027; other essential workers, from thoughtful: coefficient 0.013 to optimistic: coefficient 0.033). However, healthcare workers were more tired (coefficient: 0.009) than non-essential workers.

*RQ3: What is the relationship between experienced stressors and daily affect?*

We performed the IPW GEE linear regression of positive and negative affect ratings on seven categories of stressors, where we adjusted for the month dummy variables, the county-level COVID-19 case rates and death rates, testing for COVID, and COVID test results.

**Figure 4:**
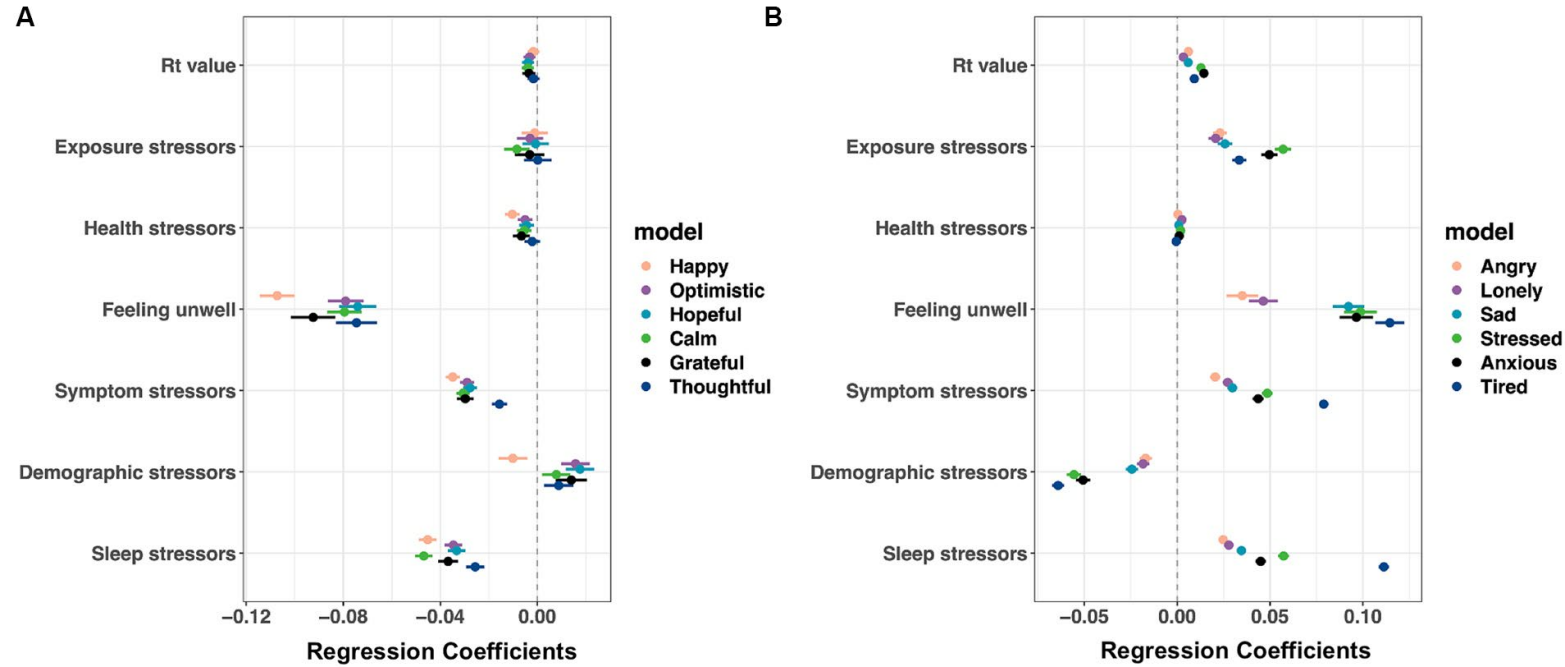
Associations between stressors and positive **(A)** and negative **(B)** affect. IPW GEE linear regression of **(A)** positive affect and **(B)** negative affect on different categories of stressors. Covariates: month dummy, county-level COVID-19 case rates and death rates, and testing for COVID and test results. Shown are estimated coefficients with 95% Cis (*n* = 3,509,982 responses from 151,049 users). For coefficients, confidence interval values, and *p*-values, see Supplementary Table S5.

As shown in Figure 4 (and Supplementary Table S5), the largest effects on positive feelings were for feeling unwell (from hopeful: coefficient −0.074 to happy: coefficient −0.107), being sleep deprived (from thoughtful: coefficient −0.026 to calm: coefficient −0.047), and COVID-19 symptoms (from thoughtful: coefficient −0.016 to happy: coefficient −0.035). Similarly, negative affect was higher among people who reported feeling unwell (from angry: coefficient 0.035 to tired: coefficient 0.114), manifesting COVID-related symptoms (from angry: coefficient 0.020 to tired: coefficient 0.079), being sleep deprived (from angry: coefficient 0.025 to tired: coefficient 0.111), and higher COVID-19 exposure (from lonely: coefficient 0.021 to stressed: coefficient 0.057).

*RQ4: What is the relationship between daily affect and preventive behavior?*

Because users were asked whether they engaged in protective behavior only if they answered “yes” to the leaving home question, we fit two separate models for the regression on staying home and the regression on facial covering and social distancing. For the regression on staying home, we fit the model for the whole sample, whereas for the regression on facial covering and social distancing, we restricted the model to observations where the users answered “yes” to the leaving home question. Protective behavior was assessed the week prior to the affect outcomes. For instance, if the user logged in on day 8 and reported their affect, we calculated the proportion of logins on days 1 through 7 where they answered “yes” to wearing facial coverings. If the user answered “yes” in more than 75% of the logins, we coded the variable “face covering for past week” as 1. Otherwise, we coded “face covering for past week” as 0. We removed observations where there were no available logins from the previous week. The IPW GEE regression tested protective behaviors over one week, predicting positive and negative affect, while month, demographics, sleep duration, whether feeling unwell, exposure to COVID-19, testing for COVID and test results, and local R_t_ values were included as covariates. The sample size for the regression on staying home was 115,241 users (3,133,829 observations), and the sample size for the regression on facial coverings and social distancing was 98,919 users (1,701,325 observations).

**Figure 5:**
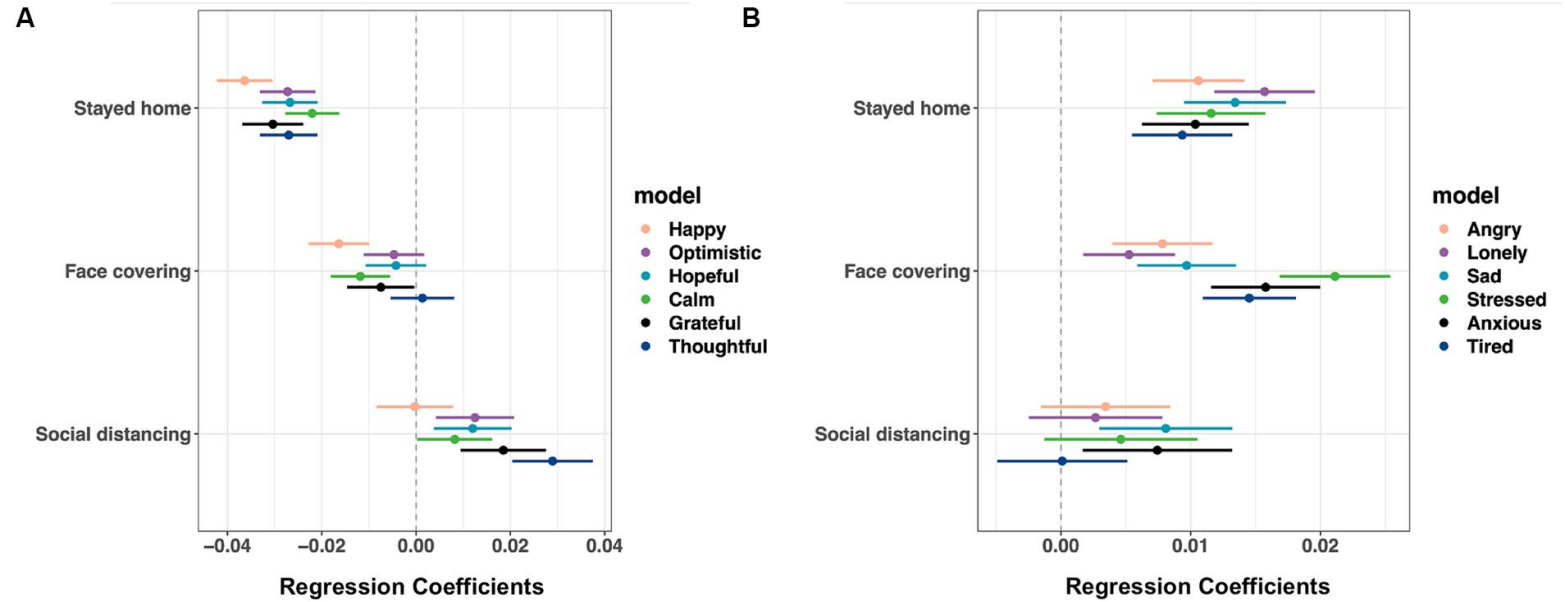
Protective behavior predicting future **(A)** positive and **(B)** negative affect. IPW GEE linear regression models were performed separately for those who responded whether they stayed at home (*n* = 3,133,829 responses from 115,241 users) and those who indicated having left home in the previous 7 days (*n* = 1,701,325 responses from 98,919 users), but the results were combined into one figure for simplicity of presentation. Covariates: month dummy variables, demographics, sleep duration, whether feeling unwell, self-reported exposure to COVID-19, testing for COVID and test results, and county-level R_t_ values. Shown are estimated coefficients with 95% Cis. For coefficients, confidence interval values, and *p*-values, see Supplementary Table S6.

Figure 5 (see Supplementary Table S6 for coefficients, confidence intervals, and *p* values) shows that those who stayed at home in the previous week reported fewer positive feelings (from calm: coefficient −0.022 to happy: coefficient −0.036) and more negative feelings (from tired: coefficient 0.009 to lonely: coefficient 0.016) the following week. Users who wore facial coverings reported lower happiness (coefficient −0.016), calm (coefficient −0.012), and gratitude (coefficient −0.007) and higher negative affect (from lonely: coefficient 0.005 to stressed: coefficient 0.021).

Those who practiced social distancing reported higher anxiety (coefficient 0.007) and sadness (coefficient 0.008) but higher positive affect (from calm: coefficient 0.008 to thoughtful: coefficient 0.029).

To study whether users’ affect predicted their protective behaviors, we fit GEE logistic regression of the three protective behaviors as outcomes and users’ affect during the prior week as the predictors. Specifically, for each report of protective behavior, we collected the affect ratings from one week prior and the averages of each of the 12 affect scores as the predictors. Observations with no affect scores available from the prior week were removed.

Three separate logistic regression models (staying home, facial covering, and social distancing respectively) were fit, with the outcome coded as 1 if the user took the protective behavior or 0 otherwise. The analysis of the staying-at-home protective behavior used the full sample, with 115,241 users (3,133,829 observations). The analyses for facial covering and social distancing were restricted to observations where the users answered “yes” to the leaving home question, and the sample size was 105,447 users (1,821,042 observations). The GEE logistic regression models were fit using the average affect scores, demographics, month, sleep duration, whether feeling unwell, getting tested for COVID, and test results as covariates.

**Figure 6:**
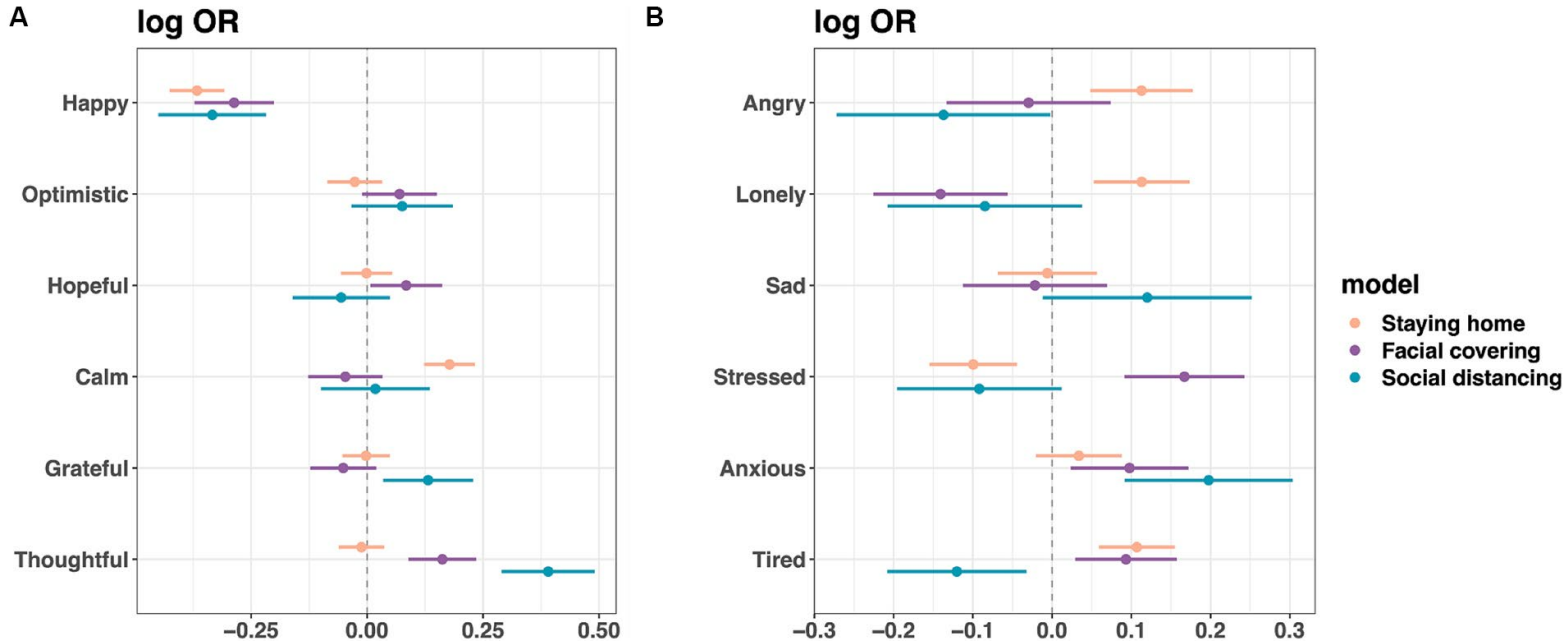
Positive affect **(A)** and negative affect **(B)** predicting future protective behavior. Three GEE logistic regression models were fit to predict staying home (*n* = 3,133,829 responses from 115,241 users), wearing facial covering, and social distancing (both *n* = 1,821,042 responses from 105,447 users) using average scores of affect variables in the previous 7 days. Covariates: demographics, month dummy variables, sleep duration, whether feeling unwell, and self-reported testing for COVID and test results. Shown are log ORs and the corresponding 95% CIs. For OR values, confidence interval values, and *p*-values, see Supplementary Table S7.

As shown in Figure 6 (see Supplementary Table S7 for odds ratios, confidence intervals, and *p* values), people who felt happier in the prior week were less likely to practice protective behavior (staying home: odds ratio (OR) = 0.693, facial covering: OR = 0.751, and social distancing: OR = 0.716), whereas those who were more grateful (OR = 1.140) or thoughtful (OR = 1.478) were more likely to practice social distancing. Those who felt more stressed (OR = 1.182), anxious (OR = 1.103), or tired (OR = 1.098) in the prior week were more likely to wear facial coverings, while those who felt angrier (OR = 1.120), lonelier (OR = 1.120), or more tired (OR = 1.113) were more likely to stay home.

## Discussion

Using data from the HWF app, we showed that between May/June 2020 and February 2021 people in the United States experienced more positive than negative affect. Throughout the pandemic, people tended to experience more positive than negative affect. Positive affect increased, and negative affect decreased over time. These findings complement research on clinically relevant outcomes (Luo et al., 2020; Ren et al., 2020; Wu et al., 2020; Bridgland et al., 2021) by offering insight into both positive and negative affect, which can inform future interventions about increasing positive affect and ameliorating negative affect.

Affect balance (ratio of positive to negative affect) is an aspect of subjective well-being (Bradburn, 1969; Diener et al., 1995), which is related to measures of mental health (Lacomba-Trejo et al., 2022) and physical health (e.g., pain; Toussaint et al., 2014). Moreover, changes in affect balance predict improvement of depression after therapy (Schwartz et al., 2002) and changes in work and relationship satisfaction and overall mental health (Schutte, 2014), with emotion regulation as a suggested mechanism contributing to affect balance and outcomes (Sirois and Hirsch, 2015; Veilleux et al., 2020). The affect ratio can be raised by interventions that reduce negative affect or those that increase positive affect. In times of exposure to low-controllability stressors (such as the pandemic), increasing positive affect might be a more effective route toward higher affect balance. Positive affect is a psychological resource that builds other resources, including effective emotion regulation that helps recovery after stress (Tugade and Fredrickson, 2004). Interventions that increase positive affect also build resilience and decrease depressive symptoms (Sin and Lyubomirsky, 2009; Kleiman et al., 2013). Furthermore, interventions aiming to raise positive affect might have an indirect effect on decreasing negative affect too because in times of stress, the correlation between positive and negative affect is stronger than in stable times (Keyes, 2000).

Of note, observed differences were small in magnitude. For example, individuals reported an increase in happiness of 0.03 and an increase in feeling thoughtful of 0.10 on a scale of 0 to 10. However, even small differences may have practical relevance (Rosenthal and Rubin, 1982; Kelley and Preacher, 2012). This may be especially relevant in a context of major life events because even a smaller change can indicate a perceptible difference from the status quo.

Demographic differences during the pandemic mirror those from before the pandemic. Younger people and women reported more negative and less positive affect. As people age, they optimize well-being by selectively focusing on emotionally meaningful goals and regulating their emotions better (Urry and Gross, 2010; Sims et al., 2015; Carstensen et al., 2020; Burr et al., 2021). The affective benefits of being older during the pandemic are not due to denying risk. Older participants were found to have greater emotional well-being despite following news more closely than younger adults (Jurkowitz and Mitchell, 2020), perceiving a greater risk of infection, and having similar financial stress to younger adults (Carstensen et al., 2020). Vaccination rates are substantially higher in older adults, further attesting to their awareness of COVID-19 risk (CDC, 2021).

Similarly to pre-pandemic times (Terracciano et al., 2003; Crawford and Henry, 2004), women experienced more negative and less positive affect compared to men. Research on affect at work is relevant to these findings; men with children are happier at home (Brody and Hall, 2008), whereas women with children are happier at work (Larson et al., 1994). The pandemic disrupted work, adding emotional demand disproportionately on women, including childcare/homeschooling responsibilities. People with more than three children were happier, possibly because children had companions, but the available data did not allow for examination of possible mechanisms.

African American and Hispanic/Latinx participants reported more positive and less negative feelings, mirroring limited pre-pandemic findings (Consedine and Magai, 2002; Lankarani and Assari, 2017), despite greater cultural awareness of racial inequities and greater risk of COVID-19 infection. These differences might be explained by emotion socialization; African American mothers support expression of negative feelings less than White mothers, reflecting their desire to protect children from discrimination (Nelson et al., 2012).

This study did not find differences in negative affect among healthcare, other essential, and non-essential workers. However, healthcare workers reported more positive affect than non-essential workers. This is not surprising; healthcare workers reported an increased sense of meaning/purpose during the COVID-19 pandemic (Shechter et al., 2020), an aspect of psychological well-being (Kashdan et al., 2008). This is a psychological resource that can further build resilience (Kleiman et al., 2013).

As expected, exposure to stressors is related to daily affect. Some stressors – feeling unwell, COVID-19 symptoms, sleep deficits – were related to less positive and more negative affect. Inadequate sleep is especially noteworthy as both pre-pandemic research (Talbot et al., 2010) and studies during the pandemic (Franceschini et al., 2020; Shi et al., 2020; Dai et al., 2021) demonstrated its importance for mental health and well-being. Personal exposure to COVID-19 is most strongly related to stress and anxiety, likely reflecting infection concerns (McGinty et al., 2020). These results have implications for post-pandemic affect. Removing the risk of exposure will remove one source of stress and anxiety and likely reduce those feelings. However, reducing negative affect does not necessarily increase positive affect, especially in times of low disruption (Keyes, 2000, 2005). As mental health professionals help to build greater well-being post-pandemic, removal of stressors is only one part of the well-being equation. Another important part is creating circumstances that enable experiencing positive emotions and building of other psychological resources associated with flourishing.

As predicted by the feelings-as-information model (Clore et al., 1994; Schwartz, 2012), happiness predicted less preventive behavior a week later, similar to the results in Study 3 by Krekel et al. (2020). Happiness signals that “all is good,” which reduces the perceived need for preventive behaviors. Furthermore, those who are more thoughtful – indicating reflection and concern for others – are likely to socially distance and wear facial coverings. Stress predicts less staying at home but greater likelihood of wearing facial coverings. The present study does not allow testing this effect when controlling for responsibilities (e.g., work) that make it impossible to stay at home. As was the case before the pandemic (Hay et al., 2006), anxiety predicts preventive behaviors –wearing facial coverings and socially distancing.

These results have implications for public health policy/communication. Messaging focusing on concern or worry for others is most likely to be effective. This naturalistic study is thus in line with experimental research showing that inducing feelings of threat to one’s community (Capraro and Barcelo, 2020) or empathy for those most vulnerable (Pfattheicher et al., 2020) increases intentions to wear face coverings. Actions that benefit someone else (rather than oneself) have the additional effect of increasing individual well-being, creating a spiral of doing good, and creating meaningful and satisfying lives (Steger et al., 2008).

Earlier preventive behaviors also predicted later feelings. Staying at home most strongly predicts less positive and more negative affect a week later. These findings about the most restrictive protective measure reflect the importance of social relationships for emotional well-being (Diener and Seligman, 2002; Robinson and Martin, 2008; Diener et al., 2018). Wearing face coverings predicts less happiness and calmness and more negative affect (especially being tired, stressed, anxious), suggesting that masks are a concrete reminder of the pandemic exhaustion and uncertainty. Of note, it is possible that this is an example of earlier feelings predicting later feelings (e.g., anxiety predicting protective behavior and also later anxiety). Although we cannot exclude this possibility, there is theoretical reason to believe that affect influences behavior and that behavior in turn affects subsequent affect.

Although the present study has a number of strengths – the assessment of multiple positive and negative feelings, data through nine pandemic months, a large national sample – several limitations should be noted. While the sample is large, it is a sample of convenience and not representative of the U.S. population. The sample was disproportionately compose of women and underrepresented African American and Asian groups. Nevertheless, gender and ethnic/racial group differences were consistent with previous studies, offering credence to the results. Another limitation is that the data did not include questions about social or work responsibilities. For instance, experience of stress predicted less staying at home but more wearing of facial coverings. This could be due to a stress-inducing inability to work from home and a greater desire to protect oneself from infection; however, the available data did not allow to test this. Another variable that could be associated with affect was vaccination status. The data used in this study range only from May/June 2020 to February 2021, a time before vaccines became widely available. Therefore, we were unable to assess the impact of this variable.

Because of the nature of the data source – a smartphone app – and the way people typically engage with apps, the sample included a substantial dropout after the initial download, and this dropout could potentially have biased the results. The regression predicting continuing the use of the app based on earlier affect showed some significant effects. However, the effects were not systematic. Continuing participation was related to less anger but more sadness, loneliness, and anxiety, as well as less calm, but more optimism, hope, and thoughtfulness. Observed changes in positive affect (compared to the assessments at the start of the study period in May/June 2020) showed a consistent increase in all specific affect variables. With the exception of loneliness, other variables of negative affect tended to decrease through the pandemic months. Thus, it is unlikely that the affective experiences of those who dropped out systematically biased the results.

Another limitation is the lack of information about appraisals. Theoretical accounts of emotions describe them as arising from appraisals or interpretations of experiences based on novelty, causes, compatibility with individual values and social norms, situational urgency, and perceived controllability (Ellsworth and Scherer, 2003). For instance, stress is best described as an ambiguous feeling (Lazarus and Folkman, 1984; Folkman, 2013); when appraised as controllable, stressors are interpreted as a challenge, and negative health outcomes are less likely (Lazarus and Folkman, 1984; Folkman, 2013). On the other hand, if situations are appraised as not controllable and one’s resources as not matching the demands of the experience, stressors are interpreted as a threat and related to harmful health outcomes. Understanding appraisals is necessary to evaluate the health risks associated with affective experiences.

Research points to the importance of patterns of affective experiences (Moeller et al., 2018a,b). People who show profiles of both high positive and high negative affect could benefit from different interventions than those who experience primarily negative affect. While the former group could benefit most from the reduction in negative affect, the latter will also benefit from interventions that increase positive affect. As pandemic-related restrictions are lifted, activities that promote positive affect are becoming more accessible (e.g., social and cultural experiences). Future research will have to address whether this is reflected in the reduction of the number of people experiencing high negative and low positive affect. Moreover, it will be important to examine how many people move from the profile of high negative and high positive affect to predominantly high positive affect. These questions have direct implications for understanding resilience in the face of prolonged societal stressors and for our understanding of the mental health risks brought about by the pandemic.

## Conclusion

Using a national mobile application-based survey, we examined daily positive and negative affect between May/June 2020 and February 2021 (N=151,049; 3,509,982 observations). Daily affect is key to mental health and predicts physical health, yet little is known about discrete daily positive and negative affect during the pandemic. Daily reports of six positive (happy, optimistic, hopeful, calm, grateful, thoughtful) and six negative feelings (angry, lonely, sad, stressed, anxious, tired) showed that people experienced more positive than negative affect; positive affect increased, and negative affect decreased over time. Demographic differences mirrored those from before the pandemic, revealing young adults and women as being most vulnerable to negative affect. The effects of stressors were cumulative. Exercising protective behaviors predicted future affect, and affect also predicted future protective behaviors. The results point to messages focused on gratitude as likely to enhance protective behavior.

## Data availability statement

The raw data supporting the conclusions of this article will be made available by the authors, without undue reservation.

## Ethics statement

The studies involving humans were approved by Harvard University Longwood Medical Area Institutional Review Board (IRB). The studies were conducted in accordance with the local legislation and institutional requirements. The ethics committee/institutional review board waived the requirement of written informed consent for participation from the participants or the participants’ legal guardians/next of kin because data was collected from a mobile phone app. Participants were presented an option to donate their data for research. Only those who donated the data were included in the research.

## Author’s note

The study reported in this article was not preregistered. The data have not been made available on a permanent third-party archive, but researchers can apply to use the data by contacting the corresponding authors. Researchers with appropriate IRB approval and data security approval to perform research involving human subjects using the How We Feel data can apply to obtain access to the data used in the analysis.

## Author contributions

ZI and SS wrote the manuscript. SS performed data analyses and prepared figures. SL prepared supplementary tables. MB developed affect survey and ZI developed research questions. DC, RP, and BS designed and implemented the How We Feel application. BS and FZ initiated the project. MB and XL supervised all aspects of the work. All authors contributed to the article and approved the submitted version.

## Footnotes

^1^https://www.cdc.gov/coronavirus/2019-ncov/symptoms-testing/symptoms.html

^2^The HWF app was designed and promoted as a COVID-19 symptom monitoring app. In this context, the “feeling unwell” question was asked to screen for potential physical symptoms. We decided to control for responses to this question in order to assess affect and changes in affect that were not influenced by physical symptoms.
