# Supplementary material for: Daily positive and negative affect during the COVID-19 pandemic
Source: Front Psychol. 2024 Jan 8;14:1239123. doi: 10.3389/fpsyg.2023.1239123 (PMC10800618; doi:10.3389/fpsyg.2023.1239123)
Supplement: Supplementary file 1 [file Data_Sheet_1.docx]

Supplementary Materials: R codes

library(geepack)

library(dotwhisker)

library(dplyr)

library(ggsci)

library(RColorBrewer)

load("/ncf/xlin_covid/Users/sshen/dat5_02_09/dat_prot0.RData")

dat_ipw_complete <- dat_ipw_complete[!is.na(dat_ipw_complete$mean_angry),]

geefit_stayhome_emo<-geeglm(left_home_stayed_home~age_range_NEW+ gender+race_cat+regions+ month1+sleep+live_alone+income_cat+density_cat+ feeling+weekend+covid_tested+covid_positive+profession_essential_cat+number_preexisting_cat+mean_angry+mean_anxious+mean_lonely+mean_sad+mean_stressed+mean_tired+mean_calm+mean_grateful+mean_happy+mean_hopeful+mean_optimistic+mean_thoughtful, id=session_id, family=binomial(), corstr="independence", data=dat_ipw_complete)

fit_stayhome <- broom::tidy(geefit_stayhome_emo,conf.int = TRUE) %>% mutate(model = "Staying home") %>%

relabel_predictors(c("mean_angry" = "Angry",

"mean_lonely" = "Lonely",

"mean_sad" = "Sad",

"mean_stressed" = "Stressed",

"mean_anxious" = "Anxious",

"mean_tired" = "Tired",

"mean_happy" = "Happy",

"mean_optimistic" = "Optimistic",

"mean_hopeful" = "Hopeful",

"mean_calm" = "Calm",

"mean_grateful" = "Grateful",

"mean_thoughtful" = "Thoughtful"))

dat_prot1 <- dat_ipw_complete[dat_ipw_complete$protective_measures_social_distancing != 'not_asked',]

dat_prot1$protective_measures_social_distancing <- dat_prot1$protective_measures_social_distancing == 'True'

geefit_socialdist_emo<-geeglm(protective_measures_social_distancing~age_range_NEW+ gender+race_cat+regions+ month1+sleep+live_alone+income_cat+density_cat+ feeling+weekend+covid_tested+covid_positive+profession_essential_cat+number_preexisting_cat+mean_angry+mean_anxious+mean_lonely+mean_sad+mean_stressed+mean_tired+mean_calm+mean_grateful+mean_happy+mean_hopeful+mean_optimistic+mean_thoughtful, id=session_id, family=binomial(), corstr="independence", data=dat_prot1)

fit_socialdist <- broom::tidy(geefit_socialdist_emo,conf.int = TRUE) %>% mutate(model = "Social distancing") %>%

relabel_predictors(c("mean_angry" = "Angry",

"mean_lonely" = "Lonely",

"mean_sad" = "Sad",

"mean_stressed" = "Stressed",

"mean_anxious" = "Anxious",

"mean_tired" = "Tired",

"mean_happy" = "Happy",

"mean_optimistic" = "Optimistic",

"mean_hopeful" = "Hopeful",

"mean_calm" = "Calm",

"mean_grateful" = "Grateful",

"mean_thoughtful" = "Thoughtful"))

dat_prot2 <- dat_ipw_complete[!is.na(dat_ipw_complete$face_covering),]

geefit_facecover_emo<-geeglm(face_covering~age_range_NEW+ gender+race_cat+regions+ month1+sleep+live_alone+income_cat+density_cat+ feeling+weekend+covid_tested+covid_positive+profession_essential_cat+number_preexisting_cat+mean_angry+mean_anxious+mean_lonely+mean_sad+mean_stressed+mean_tired+mean_calm+mean_grateful+mean_happy+mean_hopeful+mean_optimistic+mean_thoughtful, id=session_id, family=binomial(), corstr="independence", data=dat_prot2)

fit_facecover <- broom::tidy(geefit_facecover_emo,conf.int = TRUE) %>% mutate(model = "Facial covering") %>%

relabel_predictors(c("mean_angry" = "Angry",

"mean_lonely" = "Lonely",

"mean_sad" = "Sad",

"mean_stressed" = "Stressed",

"mean_anxious" = "Anxious",

"mean_tired" = "Tired",

"mean_happy" = "Happy",

"mean_optimistic" = "Optimistic",

"mean_hopeful" = "Hopeful",

"mean_calm" = "Calm",

"mean_grateful" = "Grateful",

"mean_thoughtful" = "Thoughtful"))

fit_emo_prot_pos <- rbind(fit_stayhome[7:12,],fit_facecover[7:12,],fit_socialdist[7:12,])

{dwplot(fit_emo_prot_pos, vline = geom_vline(xintercept = 0, colour = "grey60", linetype = 2), dot_args = list(size = 2.5), whisker_args = list(size = 1))+ggtitle('log OR') +theme_bw()+theme(plot.title = element_text(face="bold"), text=element_text(size=17, face="bold"))+ scale_color_manual(values = c("#0099B4FF", "#925E9FFF", "#FDAF91FF")) }

ggsave('/ncf/xlin_covid/Users/sshen/dat5_02_09/HWF_plots/emo_prot_pos.pdf',height = 6,width = 8)

fit_emo_prot_neg <- rbind(fit_stayhome[1:6,],fit_facecover[1:6,],fit_socialdist[1:6,])

{dwplot(fit_emo_prot_neg, vline = geom_vline(xintercept = 0, colour = "grey60", linetype = 2), dot_args = list(size = 2.5), whisker_args = list(size = 1))+ggtitle('log OR') +theme_bw()+theme(plot.title = element_text(face="bold"), text=element_text(size=17, face="bold"))+ scale_color_manual(values = c("#0099B4FF", "#925E9FFF", "#FDAF91FF")) }

ggsave('/ncf/xlin_covid/Users/sshen/dat5_02_09/HWF_plots/emo_prot_neg.pdf',height = 6,width = 8)

save(fit_emo_prot_pos,fit_emo_prot_neg,file = "/ncf/xlin_covid/Users/sshen/dat5_02_09/results/fit_emo_prot.RData")

library(geepack)

library(dotwhisker)

library(dplyr)

library(ggsci)

library(RColorBrewer)

#angry, lonely, sad, stressed, anxious, tired

#angry, lonely, sad, stressed, anxious, tired

load('/ncf/xlin_covid/Users/sshen/dat5_02_09/reg_data1.RData')

dat_ipw_complete$income_cat <- relevel(dat_ipw_complete$income_cat, ref = "[0,4.49e+04)")

geefit_angry_ipw<-geeglm(emotions_angry~age_range_NEW+ gender+race_cat_new+ before_5_am+after_5_am+regions+ month1+sleep+live_alone+income_cat+density_cat+ feeling+weekend+community_exposed + household_exposed +Case_rate +Death_rate+rt_greater_than_1+covid_tested+covid_positive+household_children_count+profession_essential_cat+number_preexisting_cat, id=session_id, family=gaussian(), corstr="independence", data=dat_ipw_complete,weights=weights)

geefit_lonely_ipw<-geeglm(emotions_lonely~age_range_NEW+ gender+race_cat_new+ before_5_am+after_5_am+regions+ month1+sleep+live_alone+income_cat+density_cat+ feeling+weekend+community_exposed + household_exposed +Case_rate +Death_rate+rt_greater_than_1+covid_tested+covid_positive+household_children_count+profession_essential_cat+number_preexisting_cat, id=session_id, family=gaussian(), corstr="independence", data=dat_ipw_complete,weights=weights)

geefit_sad_ipw<-geeglm(emotions_sad~age_range_NEW+ gender+race_cat_new+ before_5_am+after_5_am+regions+ month1+sleep+live_alone+income_cat+density_cat+ feeling+weekend+community_exposed + household_exposed +Case_rate +Death_rate+rt_greater_than_1+covid_tested+covid_positive+household_children_count+profession_essential_cat+number_preexisting_cat, id=session_id, family=gaussian(), corstr="independence", data=dat_ipw_complete,weights=weights)

geefit_stressed_ipw<-geeglm(emotions_stressed~age_range_NEW+ gender+race_cat_new+ before_5_am+after_5_am+regions+ month1+sleep+live_alone+income_cat+density_cat+ feeling+weekend+community_exposed + household_exposed +Case_rate +Death_rate+rt_greater_than_1+covid_tested+covid_positive+household_children_count+profession_essential_cat+number_preexisting_cat, id=session_id, family=gaussian(), corstr="independence", data=dat_ipw_complete,weights=weights)

geefit_tired_ipw<-geeglm(emotions_tired~age_range_NEW+ gender+race_cat_new+ before_5_am+after_5_am+regions+ month1+sleep+live_alone+income_cat+density_cat+ feeling+weekend+community_exposed + household_exposed +Case_rate +Death_rate+rt_greater_than_1+covid_tested+covid_positive+household_children_count+profession_essential_cat+number_preexisting_cat, id=session_id, family=gaussian(), corstr="independence", data=dat_ipw_complete,weights=weights)

geefit_anxious_ipw<-geeglm(emotions_anxious~age_range_NEW+ gender+race_cat_new+ before_5_am+after_5_am+regions+ month1+sleep+live_alone+income_cat+density_cat+ feeling+weekend+community_exposed + household_exposed +Case_rate +Death_rate+rt_greater_than_1+covid_tested+covid_positive+household_children_count+profession_essential_cat+number_preexisting_cat, id=session_id, family=gaussian(), corstr="independence", data=dat_ipw_complete,weights=weights)

#geefit_angry<-geeglm(emotions_angry~age_range_NEW+ gender+race_cat_new+ before_5_am+after_5_am+regions+ month1+sleep+live_alone+income_cat+density_cat+ feeling+weekend+community_exposed + household_exposed +Case_rate +Death_rate + rt_greater_than_1 +covid_tested+covid_positive+household_children_count+profession_essential_cat+number_preexisting_cat, id=session_id, family=gaussian(), corstr="independence", data=dat_ipw_complete)

brackets <- list(c("18-30","Age range 30-45", "Age range 80+" ),

c("Male", "Female", "Other gender"),

c("White", "African American","Other Race"),

c("Northeast", "Midwest","West"),

c("May-June, 2020","Jul, 2020","Feb, 2021"),

c("7 to 8 hrs", "Sleep less than 5", "Sleep 11 or more"),

c("44.9k-65.7k","Median income 0-44.9k","Median income 65.7k+"),

c("0-150","Density 150-1000", "Density 1000+"),

c("0","Household children count 1","Household children count 3+"),

c("Profession: \nNon-essential","Healthcare professional", "Other essential workers"),

c("0","No. preexisting conditions 1", "No. preexisting conditions 4+")

)

brackets_demo1 <- list(c("Age range: \n18-30","30-45", "80+" ),

c("Gender: \nMale", "Female", "Other"),

c("Race/Ethnicity: \nWhite", "African American"," Other"),

c("Region \nNortheast", "Midwest","West")

)

brackets_demo2 <- list(c("Median income: \n0-44.9k","44.9k-65.7k","65.7k+"),

c("Density: \n0-150","150-1000", "1000+"),

c("No. of children: 0","1","5+"),

c("Profession: \nNon-essential","Healthcare professional", "Other essential workers")

)

brackets_time <- list(#c("18-30","Age range 30-45", "Age range 80+" ),

#c("Male", "Female", "Other gender"),

#c("White", "African American","Other Race"),

#c("Northeast", "Midwest","West"),

c("May-June, 2020","Jul, 2020","Feb, 2021")

#c("7 to 8 hrs", "Sleep less than 5", "Sleep 11 or more"),

#c("44.9k-65.7k","Median income 0-44.9k","Median income 65.7k+"),

#c("0-150","Density 150-1000", "Density 1000+"),

#c("0","Household children count 1","Household children count 3+"),

#c("Non-essential","Healthcare professional", "Other essential workers"),

#c("0","No. preexisting conditions 1", "No. preexisting conditions 4+")

)

brackets_stressor <- list(#c("Age range: \n 18-30","Age range 30-45", "Age range 80+" ),

#c("Male", "Female", "Other gender"),

#c("White", "African American","Other Race"),

#c("Northeast", "Midwest","West"),

#c("May-June, 2020","Jul, 2020","Feb, 2021"),

#c("Sleep: \n7 to 8 hrs", "less than 5", "11 or more"),

#c("44.9k-65.7k","Median income 0-44.9k","Median income 65.7k+"),

#c("0-150","Density 150-1000", "Density 1000+"),

c("Household children count: \n0","1","3+"),

c("Non-essential","Healthcare professional", "Other essential workers"),

c("No. preexisting conditions: \n0"," 1", " 4+")

)

fit_angry <- broom::tidy(geefit_angry_ipw,conf.int = TRUE) %>% mutate(model = "Angry") %>%

relabel_predictors(c("genderfemale" = "Female",

"genderother" = "Other",

"age_range_NEW[30,45)" = "30-45",

"age_range_NEW[45,60)" = "45-60",

"age_range_NEW[60,80)" = "60-80",

"age_range_NEW[80+)" = "80+",

"race_cat_newafrican_american" = "African American",

"race_cat_newasian" = "Asian",

"race_cat_newhispanic_latino" = "Hispanic/Latinx" ,

"race_cat_newhawaiian_or_islander" = "Hawaiian/Islander" ,

"race_cat_newnative" = "American Indian/Alaska Native" ,

"race_cat_newmultiracial" = "Multiracial",

"race_cat_newother" = " Other",

"before_5_am" = "Before 5 a.m.",

"after_5_am" = "After 5 a.m.",

"regionsMidwest" = "Midwest",

"regionsSouth" = "South",

"regionsWest" = "West",

"month120-05_6" = "May-June, 2020",

#"month20-06" = "Jun, 2020",

"month120-07" = "Jul, 2020",

"month120-08" = "Aug, 2020",

"month120-09" = "Sep, 2020",

"month120-10" = "Oct, 2020",

"month120-11" = "Nov, 2020",

"month120-12" = "Dec, 2020",

"month121-01" = "Jan, 2021",

"month121-02" = "Feb, 2021",

"sleepless_than_5" = "less than 5",

"sleep5_to_6_hours" = "5 to 6 hrs",

"sleep9_to_10_hours" = "9 to 10 hrs",

"sleep11_or_more_hours" = "11 or more",

"live_aloneTRUE" = "Live Alone",

"income_cat[0,4.49e+04)" = "0-44.9k",

"income_cat[4.49e+04,6.57e+04)" = "44.9k-65.7k",

"income_cat[6.57e+04,Inf)" = "65.7k+",

"density_cat[150,1e+03)" = "150-1000",

"density_cat[1e+03,Inf)" = "1000+",

"feelingnot_well" = "Feeling unwell",

"weekendTRUE" = "Weekend",

"community_exposedTRUE" = "Community exposed",

"household_exposedTRUE" = "Household exposed",

"Case_rate" = "Case rate",

"Death_rate" = "Death rate",

"rt_greater_than_1TRUE" = "Rt > 1",

"covid_testedTRUE" = "Tested for COVID-19",

"covid_positiveTRUE" = "Positive for COVID-19",

"household_children_count1" = "1",

"household_children_count2" = "2",

"household_children_count3" = "3",

"household_children_count4" = "4",

"household_children_count5" = "5+",

"profession_essential_cathealthcare" = "Healthcare professional",

"profession_essential_catother_essential" = "Other essential workers",

"number_preexisting_cat1" = " 1",

"number_preexisting_cat2" = " 2",

"number_preexisting_cat3"= " 3",

"number_preexisting_cat4+" = " 4+"))

fit_stressed <- broom::tidy(geefit_stressed_ipw,conf.int = TRUE) %>% mutate(model = "Stressed") %>%

relabel_predictors(c("genderfemale" = "Female",

"genderother" = "Other",

"age_range_NEW[30,45)" = "30-45",

"age_range_NEW[45,60)" = "45-60",

"age_range_NEW[60,80)" = "60-80",

"age_range_NEW[80+)" = "80+",

"race_cat_newafrican_american" = "African American",

"race_cat_newasian" = "Asian",

"race_cat_newhispanic_latino" = "Hispanic/Latinx" ,

"race_cat_newhawaiian_or_islander" = "Hawaiian/Islander" ,

"race_cat_newnative" = "American Indian/Alaska Native" ,

"race_cat_newmultiracial" = "Multiracial",

"race_cat_newother" = " Other",

"before_5_am" = "Before 5 a.m.",

"after_5_am" = "After 5 a.m.",

"regionsMidwest" = "Midwest",

"regionsSouth" = "South",

"regionsWest" = "West",

"month120-05_6" = "May-June, 2020",

#"month20-06" = "Jun, 2020",

"month120-07" = "Jul, 2020",

"month120-08" = "Aug, 2020",

"month120-09" = "Sep, 2020",

"month120-10" = "Oct, 2020",

"month120-11" = "Nov, 2020",

"month120-12" = "Dec, 2020",

"month121-01" = "Jan, 2021",

"month121-02" = "Feb, 2021",

"sleepless_than_5" = "less than 5",

"sleep5_to_6_hours" = "5 to 6 hrs",

"sleep9_to_10_hours" = "9 to 10 hrs",

"sleep11_or_more_hours" = "11 or more",

"live_aloneTRUE" = "Live Alone",

"income_cat[0,4.49e+04)" = "0-44.9k",

"income_cat[4.49e+04,6.57e+04)" = "44.9k-65.7k",

"income_cat[6.57e+04,Inf)" = "65.7k+",

"density_cat[150,1e+03)" = "150-1000",

"density_cat[1e+03,Inf)" = "1000+",

"feelingnot_well" = "Feeling unwell",

"weekendTRUE" = "Weekend",

"community_exposedTRUE" = "Community exposed",

"household_exposedTRUE" = "Household exposed",

"Case_rate" = "Case rate",

"Death_rate" = "Death rate",

"rt_greater_than_1TRUE" = "Rt > 1",

"covid_testedTRUE" = "Tested for COVID-19",

"covid_positiveTRUE" = "Positive for COVID-19",

"household_children_count1" = "1",

"household_children_count2" = "2",

"household_children_count3" = "3",

"household_children_count4" = "4",

"household_children_count5" = "5+",

"profession_essential_cathealthcare" = "Healthcare professional",

"profession_essential_catother_essential" = "Other essential workers",

"number_preexisting_cat1" = " 1",

"number_preexisting_cat2" = " 2",

"number_preexisting_cat3"= " 3",

"number_preexisting_cat4+" = " 4+"))

fit_tired <- broom::tidy(geefit_tired_ipw,conf.int = TRUE) %>% mutate(model = "Tired") %>%

relabel_predictors(c("genderfemale" = "Female",

"genderother" = "Other",

"age_range_NEW[30,45)" = "30-45",

"age_range_NEW[45,60)" = "45-60",

"age_range_NEW[60,80)" = "60-80",

"age_range_NEW[80+)" = "80+",

"race_cat_newafrican_american" = "African American",

"race_cat_newasian" = "Asian",

"race_cat_newhispanic_latino" = "Hispanic/Latinx" ,

"race_cat_newhawaiian_or_islander" = "Hawaiian/Islander" ,

"race_cat_newnative" = "American Indian/Alaska Native" ,

"race_cat_newmultiracial" = "Multiracial",

"race_cat_newother" = " Other",

"before_5_am" = "Before 5 a.m.",

"after_5_am" = "After 5 a.m.",

"regionsMidwest" = "Midwest",

"regionsSouth" = "South",

"regionsWest" = "West",

"month120-05_6" = "May-June, 2020",

#"month20-06" = "Jun, 2020",

"month120-07" = "Jul, 2020",

"month120-08" = "Aug, 2020",

"month120-09" = "Sep, 2020",

"month120-10" = "Oct, 2020",

"month120-11" = "Nov, 2020",

"month120-12" = "Dec, 2020",

"month121-01" = "Jan, 2021",

"month121-02" = "Feb, 2021",

"sleepless_than_5" = "less than 5",

"sleep5_to_6_hours" = "5 to 6 hrs",

"sleep9_to_10_hours" = "9 to 10 hrs",

"sleep11_or_more_hours" = "11 or more",

"live_aloneTRUE" = "Live Alone",

"income_cat[0,4.49e+04)" = "0-44.9k",

"income_cat[4.49e+04,6.57e+04)" = "44.9k-65.7k",

"income_cat[6.57e+04,Inf)" = "65.7k+",

"density_cat[150,1e+03)" = "150-1000",

"density_cat[1e+03,Inf)" = "1000+",

"feelingnot_well" = "Feeling unwell",

"weekendTRUE" = "Weekend",

"community_exposedTRUE" = "Community exposed",

"household_exposedTRUE" = "Household exposed",

"Case_rate" = "Case rate",

"Death_rate" = "Death rate",

"rt_greater_than_1TRUE" = "Rt > 1",

"covid_testedTRUE" = "Tested for COVID-19",

"covid_positiveTRUE" = "Positive for COVID-19",

"household_children_count1" = "1",

"household_children_count2" = "2",

"household_children_count3" = "3",

"household_children_count4" = "4",

"household_children_count5" = "5+",

"profession_essential_cathealthcare" = "Healthcare professional",

"profession_essential_catother_essential" = "Other essential workers",

"number_preexisting_cat1" = " 1",

"number_preexisting_cat2" = " 2",

"number_preexisting_cat3"= " 3",

"number_preexisting_cat4+" = " 4+"))

fit_anxious <- broom::tidy(geefit_anxious_ipw,conf.int = TRUE) %>% mutate(model = "Anxious") %>%

relabel_predictors(c("genderfemale" = "Female",

"genderother" = "Other",

"age_range_NEW[30,45)" = "30-45",

"age_range_NEW[45,60)" = "45-60",

"age_range_NEW[60,80)" = "60-80",

"age_range_NEW[80+)" = "80+",

"race_cat_newafrican_american" = "African American",

"race_cat_newasian" = "Asian",

"race_cat_newhispanic_latino" = "Hispanic/Latinx" ,

"race_cat_newhawaiian_or_islander" = "Hawaiian/Islander" ,

"race_cat_newnative" = "American Indian/Alaska Native" ,

"race_cat_newmultiracial" = "Multiracial",

"race_cat_newother" = " Other",

"before_5_am" = "Before 5 a.m.",

"after_5_am" = "After 5 a.m.",

"regionsMidwest" = "Midwest",

"regionsSouth" = "South",

"regionsWest" = "West",

"month120-05_6" = "May-June, 2020",

#"month20-06" = "Jun, 2020",

"month120-07" = "Jul, 2020",

"month120-08" = "Aug, 2020",

"month120-09" = "Sep, 2020",

"month120-10" = "Oct, 2020",

"month120-11" = "Nov, 2020",

"month120-12" = "Dec, 2020",

"month121-01" = "Jan, 2021",

"month121-02" = "Feb, 2021",

"sleepless_than_5" = "less than 5",

"sleep5_to_6_hours" = "5 to 6 hrs",

"sleep9_to_10_hours" = "9 to 10 hrs",

"sleep11_or_more_hours" = "11 or more",

"live_aloneTRUE" = "Live Alone",

"income_cat[0,4.49e+04)" = "0-44.9k",

"income_cat[4.49e+04,6.57e+04)" = "44.9k-65.7k",

"income_cat[6.57e+04,Inf)" = "65.7k+",

"density_cat[150,1e+03)" = "150-1000",

"density_cat[1e+03,Inf)" = "1000+",

"feelingnot_well" = "Feeling unwell",

"weekendTRUE" = "Weekend",

"community_exposedTRUE" = "Community exposed",

"household_exposedTRUE" = "Household exposed",

"Case_rate" = "Case rate",

"Death_rate" = "Death rate",

"rt_greater_than_1TRUE" = "Rt > 1",

"covid_testedTRUE" = "Tested for COVID-19",

"covid_positiveTRUE" = "Positive for COVID-19",

"household_children_count1" = "1",

"household_children_count2" = "2",

"household_children_count3" = "3",

"household_children_count4" = "4",

"household_children_count5" = "5+",

"profession_essential_cathealthcare" = "Healthcare professional",

"profession_essential_catother_essential" = "Other essential workers",

"number_preexisting_cat1" = " 1",

"number_preexisting_cat2" = " 2",

"number_preexisting_cat3"= " 3",

"number_preexisting_cat4+" = " 4+"))

fit_sad <- broom::tidy(geefit_sad_ipw,conf.int = TRUE) %>% mutate(model = "Sad") %>%

relabel_predictors(c("genderfemale" = "Female",

"genderother" = "Other",

"age_range_NEW[30,45)" = "30-45",

"age_range_NEW[45,60)" = "45-60",

"age_range_NEW[60,80)" = "60-80",

"age_range_NEW[80+)" = "80+",

"race_cat_newafrican_american" = "African American",

"race_cat_newasian" = "Asian",

"race_cat_newhispanic_latino" = "Hispanic/Latinx" ,

"race_cat_newhawaiian_or_islander" = "Hawaiian/Islander" ,

"race_cat_newnative" = "American Indian/Alaska Native" ,

"race_cat_newmultiracial" = "Multiracial",

"race_cat_newother" = " Other",

"before_5_am" = "Before 5 a.m.",

"after_5_am" = "After 5 a.m.",

"regionsMidwest" = "Midwest",

"regionsSouth" = "South",

"regionsWest" = "West",

"month120-05_6" = "May-June, 2020",

#"month20-06" = "Jun, 2020",

"month120-07" = "Jul, 2020",

"month120-08" = "Aug, 2020",

"month120-09" = "Sep, 2020",

"month120-10" = "Oct, 2020",

"month120-11" = "Nov, 2020",

"month120-12" = "Dec, 2020",

"month121-01" = "Jan, 2021",

"month121-02" = "Feb, 2021",

"sleepless_than_5" = "less than 5",

"sleep5_to_6_hours" = "5 to 6 hrs",

"sleep9_to_10_hours" = "9 to 10 hrs",

"sleep11_or_more_hours" = "11 or more",

"live_aloneTRUE" = "Live Alone",

"income_cat[0,4.49e+04)" = "0-44.9k",

"income_cat[4.49e+04,6.57e+04)" = "44.9k-65.7k",

"income_cat[6.57e+04,Inf)" = "65.7k+",

"density_cat[150,1e+03)" = "150-1000",

"density_cat[1e+03,Inf)" = "1000+",

"feelingnot_well" = "Feeling unwell",

"weekendTRUE" = "Weekend",

"community_exposedTRUE" = "Community exposed",

"household_exposedTRUE" = "Household exposed",

"Case_rate" = "Case rate",

"Death_rate" = "Death rate",

"rt_greater_than_1TRUE" = "Rt > 1",

"covid_testedTRUE" = "Tested for COVID-19",

"covid_positiveTRUE" = "Positive for COVID-19",

"household_children_count1" = "1",

"household_children_count2" = "2",

"household_children_count3" = "3",

"household_children_count4" = "4",

"household_children_count5" = "5+",

"profession_essential_cathealthcare" = "Healthcare professional",

"profession_essential_catother_essential" = "Other essential workers",

"number_preexisting_cat1" = " 1",

"number_preexisting_cat2" = " 2",

"number_preexisting_cat3"= " 3",

"number_preexisting_cat4+" = " 4+"))

fit_lonely <- broom::tidy(geefit_lonely_ipw,conf.int = TRUE) %>% mutate(model = "Lonely") %>%

relabel_predictors(c("genderfemale" = "Female",

"genderother" = "Other",

"age_range_NEW[30,45)" = "30-45",

"age_range_NEW[45,60)" = "45-60",

"age_range_NEW[60,80)" = "60-80",

"age_range_NEW[80+)" = "80+",

"race_cat_newafrican_american" = "African American",

"race_cat_newasian" = "Asian",

"race_cat_newhispanic_latino" = "Hispanic/Latinx" ,

"race_cat_newhawaiian_or_islander" = "Hawaiian/Islander" ,

"race_cat_newnative" = "American Indian/Alaska Native" ,

"race_cat_newmultiracial" = "Multiracial",

"race_cat_newother" = " Other",

"before_5_am" = "Before 5 a.m.",

"after_5_am" = "After 5 a.m.",

"regionsMidwest" = "Midwest",

"regionsSouth" = "South",

"regionsWest" = "West",

"month120-05_6" = "May-June, 2020",

#"month20-06" = "Jun, 2020",

"month120-07" = "Jul, 2020",

"month120-08" = "Aug, 2020",

"month120-09" = "Sep, 2020",

"month120-10" = "Oct, 2020",

"month120-11" = "Nov, 2020",

"month120-12" = "Dec, 2020",

"month121-01" = "Jan, 2021",

"month121-02" = "Feb, 2021",

"sleepless_than_5" = "less than 5",

"sleep5_to_6_hours" = "5 to 6 hrs",

"sleep9_to_10_hours" = "9 to 10 hrs",

"sleep11_or_more_hours" = "11 or more",

"live_aloneTRUE" = "Live Alone",

"income_cat[0,4.49e+04)" = "0-44.9k",

"income_cat[4.49e+04,6.57e+04)" = "44.9k-65.7k",

"income_cat[6.57e+04,Inf)" = "65.7k+",

"density_cat[150,1e+03)" = "150-1000",

"density_cat[1e+03,Inf)" = "1000+",

"feelingnot_well" = "Feeling unwell",

"weekendTRUE" = "Weekend",

"community_exposedTRUE" = "Community exposed",

"household_exposedTRUE" = "Household exposed",

"Case_rate" = "Case rate",

"Death_rate" = "Death rate",

"rt_greater_than_1TRUE" = "Rt > 1",

"covid_testedTRUE" = "Tested for COVID-19",

"covid_positiveTRUE" = "Positive for COVID-19",

"household_children_count1" = "1",

"household_children_count2" = "2",

"household_children_count3" = "3",

"household_children_count4" = "4",

"household_children_count5" = "5+",

"profession_essential_cathealthcare" = "Healthcare professional",

"profession_essential_catother_essential" = "Other essential workers",

"number_preexisting_cat1" = " 1",

"number_preexisting_cat2" = " 2",

"number_preexisting_cat3"= " 3",

"number_preexisting_cat4+" = " 4+"))

demo_ind1 <- c(1:13,16:18)

demo_ind2 <- c(31:35,45:51)

time_ind <- c(19:26)

stressor_ind <- c(29,36:51)

fit_demo1 <- rbind(fit_angry[demo_ind1,],fit_lonely[demo_ind1,],fit_sad[demo_ind1,],fit_stressed[demo_ind1,],fit_anxious[demo_ind1,],fit_tired[demo_ind1,])

{dwplot(fit_demo1, vline = geom_vline(xintercept = 0, colour = "grey60", linetype = 2),dot_args = list(size = 2.5), whisker_args = list(size = 1))+theme_bw()+theme(plot.title = element_text(face="bold"), text=element_text(size=25, face="bold"))+ scale_color_manual(values = c( "#00468BFF", "black", "#42B540FF", "#0099B4FF", "#925E9FFF", "#FDAF91FF"))}%>%

add_brackets(brackets_demo1, fontSize = 1.5, face="bold")

ggsave('/ncf/xlin_covid/Users/sshen/dat5_02_09/HWF_plots/neg_demo1.pdf',height = 15,width = 15)

fit_demo2 <- rbind(fit_angry[demo_ind2,],fit_lonely[demo_ind2,],fit_sad[demo_ind2,],fit_stressed[demo_ind2,],fit_anxious[demo_ind2,],fit_tired[demo_ind2,])

{dwplot(fit_demo2, vline = geom_vline(xintercept = 0, colour = "grey60", linetype = 2), dot_args = list(size = 2.5), whisker_args = list(size = 1))+theme_bw()+theme(plot.title = element_text(face="bold"), text=element_text(size=25, face="bold"))+ scale_color_manual(values = c( "#00468BFF", "black", "#42B540FF", "#0099B4FF", "#925E9FFF", "#FDAF91FF"))}%>%

add_brackets(brackets_demo2, fontSize = 1.5, face="bold")

ggsave('/ncf/xlin_covid/Users/sshen/dat5_02_09/HWF_plots/neg_demo2.pdf',height = 15,width = 15)

fit_time <- rbind(fit_angry[time_ind,],fit_lonely[time_ind,],fit_sad[time_ind,],fit_stressed[time_ind,],fit_anxious[time_ind,],fit_tired[time_ind,])

{dwplot(fit_time, vline = geom_vline(xintercept = 0, colour = "grey60", linetype = 2), dot_args = list(size = 2.5), whisker_args = list(size = 1))+theme_bw()+theme(plot.title = element_text(face="bold"), text=element_text(size=18, face="bold"))+ scale_color_manual(values = c( "#00468BFF", "black", "#42B540FF", "#0099B4FF", "#925E9FFF", "#FDAF91FF"))}%>%

add_brackets(brackets_time, fontSize = 1.2, face="bold")

ggsave('/ncf/xlin_covid/Users/sshen/dat5_02_09/HWF_plots/neg_time.pdf',height = 8,width = 10)

save(fit_demo1, fit_demo2,fit_time,file = "/ncf/xlin_covid/Users/sshen/dat5_02_09/results/fit_demo_time_neg.RData")

#{dwplot(rbind(fit_angry[stressor_ind,],fit_lonely[stressor_ind,],fit_sad[stressor_ind,],fit_stressed[stressor_ind,],fit_anxious[stressor_ind,],fit_tired[stressor_ind,]), vline = geom_vline(xintercept = 0, colour = "grey60", linetype = 2))+theme_bw()+theme(plot.title = element_text(face="bold"))+ scale_color_manual(values = c( "#FC8D62", "#8DA0CB", "#E78AC3", "#A6D854", "#FFD92F", "#E5C494", "#B3B3B3"))}%>%

# add_brackets(brackets_stressor)

#ggsave('/ncf/xlin_covid/Users/sshen/dat5_02_09/HWF_plots/pos_stressor.png',height = 10,width = 8)

library(geepack)

library(dotwhisker)

library(dplyr)

#angry, lonely, sad, stressed, anxious, tired

#happy, lonely, sad, stressed, anxious, tired

load('/ncf/xlin_covid/Users/sshen/dat5_02_09/reg_data1.RData')

dat5_US_ipw$income_cat <- relevel(dat5_US_ipw$income_cat, ref = "[0,4.49e+04)")

geefit_obv<-geeglm(observed~age_range_NEW+ gender+race_cat+ regions+ date_centered+time_transformed+sleep+live_alone+income_cat+density_cat+ feeling + community_exposed + household_exposed + positiveIncrease + deathIncrease+positive_percapita+death_percapita, id=session_id, family=binomial(), corstr="independence", data=dat5_US_ipw)

save(geefit_obv,file="/ncf/xlin_covid/Users/sshen/dat5_02_09/gee_obv.RData")

library(geepack)

library(dotwhisker)

library(dplyr)

library(ggsci)

library(RColorBrewer)

#angry, lonely, sad, stressed, anxious, tired

#happy, optimistic, hopeful, calm, grateful, thoughtful

load('/ncf/xlin_covid/Users/sshen/dat5_02_09/reg_data1.RData')

dat_ipw_complete$income_cat <- relevel(dat_ipw_complete$income_cat, ref = "[0,4.49e+04)")

geefit_happy_ipw<-geeglm(emotions_happy~age_range_NEW+ gender+race_cat_new+ before_5_am+after_5_am+regions+ month1+sleep+live_alone+income_cat+density_cat+ feeling+weekend+community_exposed + household_exposed +Case_rate +Death_rate+rt_greater_than_1+covid_tested+covid_positive+household_children_count+profession_essential_cat+number_preexisting_cat, id=session_id, family=gaussian(), corstr="independence", data=dat_ipw_complete,weights=weights)

geefit_optimistic_ipw<-geeglm(emotions_optimistic~age_range_NEW+ gender+race_cat_new+ before_5_am+after_5_am+regions+ month1+sleep+live_alone+income_cat+density_cat+ feeling+weekend+community_exposed + household_exposed +Case_rate +Death_rate+rt_greater_than_1+covid_tested+covid_positive+household_children_count+profession_essential_cat+number_preexisting_cat, id=session_id, family=gaussian(), corstr="independence", data=dat_ipw_complete,weights=weights)

geefit_hopeful_ipw<-geeglm(emotions_hopeful~age_range_NEW+ gender+race_cat_new+ before_5_am+after_5_am+regions+ month1+sleep+live_alone+income_cat+density_cat+ feeling+weekend+community_exposed + household_exposed +Case_rate +Death_rate+rt_greater_than_1+covid_tested+covid_positive+household_children_count+profession_essential_cat+number_preexisting_cat, id=session_id, family=gaussian(), corstr="independence", data=dat_ipw_complete,weights=weights)

geefit_calm_ipw<-geeglm(emotions_calm~age_range_NEW+ gender+race_cat_new+ before_5_am+after_5_am+regions+ month1+sleep+live_alone+income_cat+density_cat+ feeling+weekend+community_exposed + household_exposed +Case_rate +Death_rate+rt_greater_than_1+covid_tested+covid_positive+household_children_count+profession_essential_cat+number_preexisting_cat, id=session_id, family=gaussian(), corstr="independence", data=dat_ipw_complete,weights=weights)

geefit_thoughtful_ipw<-geeglm(emotions_thoughtful~age_range_NEW+ gender+race_cat_new+ before_5_am+after_5_am+regions+ month1+sleep+live_alone+income_cat+density_cat+ feeling+weekend+community_exposed + household_exposed +Case_rate +Death_rate+rt_greater_than_1+covid_tested+covid_positive+household_children_count+profession_essential_cat+number_preexisting_cat, id=session_id, family=gaussian(), corstr="independence", data=dat_ipw_complete,weights=weights)

geefit_grateful_ipw<-geeglm(emotions_grateful~age_range_NEW+ gender+race_cat_new+ before_5_am+after_5_am+regions+ month1+sleep+live_alone+income_cat+density_cat+ feeling+weekend+community_exposed + household_exposed +Case_rate +Death_rate+rt_greater_than_1+covid_tested+covid_positive+household_children_count+profession_essential_cat+number_preexisting_cat, id=session_id, family=gaussian(), corstr="independence", data=dat_ipw_complete,weights=weights)

#geefit_happy<-geeglm(emotions_happy~age_range_NEW+ gender+race_cat_new+ before_5_am+after_5_am+regions+ month1+sleep+live_alone+income_cat+density_cat+ feeling+weekend+community_exposed + household_exposed +Case_rate +Death_rate + rt_greater_than_1 +covid_tested+covid_positive+household_children_count+profession_essential_cat+number_preexisting_cat, id=session_id, family=gaussian(), corstr="independence", data=dat_ipw_complete)

brackets <- list(c("18-30","Age range 30-45", "Age range 80+" ),

c("Male", "Female", "Other gender"),

c("White", "African American","Other Race"),

c("Northeast", "Midwest","West"),

c("May-June, 2020","Jul, 2020","Feb, 2021"),

c("7 to 8 hrs", "Sleep less than 5", "Sleep 11 or more"),

c("44.9k-65.7k","Median income 0-44.9k","Median income 65.7k+"),

c("0-150","Density 150-1000", "Density 1000+"),

c("0","Household children count 1","Household children count 3+"),

c("Profession: \nNon-essential","Healthcare professional", "Other essential workers"),

c("0","No. preexisting conditions 1", "No. preexisting conditions 4+")

)

brackets_demo1 <- list(c("Age range: \n18-30","30-45", "80+" ),

c("Gender: \nMale", "Female", "Other"),

c("Race/Ethnicity: \nWhite", "African American"," Other"),

c("Region \nNortheast", "Midwest","West")

)

brackets_demo2 <- list(c("Median income: \n0-44.9k","44.9k-65.7k","65.7k+"),

c("Density: \n0-150","150-1000", "1000+"),

c("No. of children: 0","1","5+"),

c("Profession: \nNon-essential","Healthcare professional", "Other essential workers")

)

brackets_time <- list(#c("18-30","Age range 30-45", "Age range 80+" ),

#c("Male", "Female", "Other gender"),

#c("White", "African American","Other Race"),

#c("Northeast", "Midwest","West"),

c("May-June, 2020","Jul, 2020","Feb, 2021")

#c("7 to 8 hrs", "Sleep less than 5", "Sleep 11 or more"),

#c("44.9k-65.7k","Median income 0-44.9k","Median income 65.7k+"),

#c("0-150","Density 150-1000", "Density 1000+"),

#c("0","Household children count 1","Household children count 3+"),

#c("Non-essential","Healthcare professional", "Other essential workers"),

#c("0","No. preexisting conditions 1", "No. preexisting conditions 4+")

)

brackets_stressor <- list(#c("Age range: \n 18-30","Age range 30-45", "Age range 80+" ),

#c("Male", "Female", "Other gender"),

#c("White", "African American","Other Race"),

#c("Northeast", "Midwest","West"),

#c("May-June, 2020","Jul, 2020","Feb, 2021"),

#c("Sleep: \n7 to 8 hrs", "less than 5", "11 or more"),

#c("44.9k-65.7k","Median income 0-44.9k","Median income 65.7k+"),

#c("0-150","Density 150-1000", "Density 1000+"),

c("Household children count: \n0","1","3+"),

c("Non-essential","Healthcare professional", "Other essential workers"),

c("No. preexisting conditions: \n0"," 1", " 4+")

)

fit_happy <- broom::tidy(geefit_happy_ipw,conf.int = TRUE) %>% mutate(model = "Happy") %>%

relabel_predictors(c("genderfemale" = "Female",

"genderother" = "Other",

"age_range_NEW[30,45)" = "30-45",

"age_range_NEW[45,60)" = "45-60",

"age_range_NEW[60,80)" = "60-80",

"age_range_NEW[80+)" = "80+",

"race_cat_newafrican_american" = "African American",

"race_cat_newasian" = "Asian",

"race_cat_newhispanic_latino" = "Hispanic/Latinx" ,

"race_cat_newhawaiian_or_islander" = "Hawaiian/Islander" ,

"race_cat_newnative" = "American Indian/Alaska Native" ,

"race_cat_newmultiracial" = "Multiracial",

"race_cat_newother" = " Other",

"before_5_am" = "Before 5 a.m.",

"after_5_am" = "After 5 a.m.",

"regionsMidwest" = "Midwest",

"regionsSouth" = "South",

"regionsWest" = "West",

"month120-05_6" = "May-June, 2020",

#"month20-06" = "Jun, 2020",

"month120-07" = "Jul, 2020",

"month120-08" = "Aug, 2020",

"month120-09" = "Sep, 2020",

"month120-10" = "Oct, 2020",

"month120-11" = "Nov, 2020",

"month120-12" = "Dec, 2020",

"month121-01" = "Jan, 2021",

"month121-02" = "Feb, 2021",

"sleepless_than_5" = "less than 5",

"sleep5_to_6_hours" = "5 to 6 hrs",

"sleep9_to_10_hours" = "9 to 10 hrs",

"sleep11_or_more_hours" = "11 or more",

"live_aloneTRUE" = "Live Alone",

"income_cat[0,4.49e+04)" = "0-44.9k",

"income_cat[4.49e+04,6.57e+04)" = "44.9k-65.7k",

"income_cat[6.57e+04,Inf)" = "65.7k+",

"density_cat[150,1e+03)" = "150-1000",

"density_cat[1e+03,Inf)" = "1000+",

"feelingnot_well" = "Feeling unwell",

"weekendTRUE" = "Weekend",

"community_exposedTRUE" = "Community exposed",

"household_exposedTRUE" = "Household exposed",

"Case_rate" = "Case rate",

"Death_rate" = "Death rate",

"rt_greater_than_1TRUE" = "Rt > 1",

"covid_testedTRUE" = "Tested for COVID-19",

"covid_positiveTRUE" = "Positive for COVID-19",

"household_children_count1" = "1",

"household_children_count2" = "2",

"household_children_count3" = "3",

"household_children_count4" = "4",

"household_children_count5" = "5+",

"profession_essential_cathealthcare" = "Healthcare professional",

"profession_essential_catother_essential" = "Other essential workers",

"number_preexisting_cat1" = " 1",

"number_preexisting_cat2" = " 2",

"number_preexisting_cat3"= " 3",

"number_preexisting_cat4+" = " 4+"))

fit_calm <- broom::tidy(geefit_calm_ipw,conf.int = TRUE) %>% mutate(model = "Calm") %>%

relabel_predictors(c("genderfemale" = "Female",

"genderother" = "Other",

"age_range_NEW[30,45)" = "30-45",

"age_range_NEW[45,60)" = "45-60",

"age_range_NEW[60,80)" = "60-80",

"age_range_NEW[80+)" = "80+",

"race_cat_newafrican_american" = "African American",

"race_cat_newasian" = "Asian",

"race_cat_newhispanic_latino" = "Hispanic/Latinx" ,

"race_cat_newhawaiian_or_islander" = "Hawaiian/Islander" ,

"race_cat_newnative" = "American Indian/Alaska Native" ,

"race_cat_newmultiracial" = "Multiracial",

"race_cat_newother" = " Other",

"before_5_am" = "Before 5 a.m.",

"after_5_am" = "After 5 a.m.",

"regionsMidwest" = "Midwest",

"regionsSouth" = "South",

"regionsWest" = "West",

"month120-05_6" = "May-June, 2020",

#"month20-06" = "Jun, 2020",

"month120-07" = "Jul, 2020",

"month120-08" = "Aug, 2020",

"month120-09" = "Sep, 2020",

"month120-10" = "Oct, 2020",

"month120-11" = "Nov, 2020",

"month120-12" = "Dec, 2020",

"month121-01" = "Jan, 2021",

"month121-02" = "Feb, 2021",

"sleepless_than_5" = "less than 5",

"sleep5_to_6_hours" = "5 to 6 hrs",

"sleep9_to_10_hours" = "9 to 10 hrs",

"sleep11_or_more_hours" = "11 or more",

"live_aloneTRUE" = "Live Alone",

"income_cat[0,4.49e+04)" = "0-44.9k",

"income_cat[4.49e+04,6.57e+04)" = "44.9k-65.7k",

"income_cat[6.57e+04,Inf)" = "65.7k+",

"density_cat[150,1e+03)" = "150-1000",

"density_cat[1e+03,Inf)" = "1000+",

"feelingnot_well" = "Feeling unwell",

"weekendTRUE" = "Weekend",

"community_exposedTRUE" = "Community exposed",

"household_exposedTRUE" = "Household exposed",

"Case_rate" = "Case rate",

"Death_rate" = "Death rate",

"rt_greater_than_1TRUE" = "Rt > 1",

"covid_testedTRUE" = "Tested for COVID-19",

"covid_positiveTRUE" = "Positive for COVID-19",

"household_children_count1" = "1",

"household_children_count2" = "2",

"household_children_count3" = "3",

"household_children_count4" = "4",

"household_children_count5" = "5+",

"profession_essential_cathealthcare" = "Healthcare professional",

"profession_essential_catother_essential" = "Other essential workers",

"number_preexisting_cat1" = " 1",

"number_preexisting_cat2" = " 2",

"number_preexisting_cat3"= " 3",

"number_preexisting_cat4+" = " 4+"))

fit_thoughtful <- broom::tidy(geefit_thoughtful_ipw,conf.int = TRUE) %>% mutate(model = "Thoughtful") %>%

relabel_predictors(c("genderfemale" = "Female",

"genderother" = "Other",

"age_range_NEW[30,45)" = "30-45",

"age_range_NEW[45,60)" = "45-60",

"age_range_NEW[60,80)" = "60-80",

"age_range_NEW[80+)" = "80+",

"race_cat_newafrican_american" = "African American",

"race_cat_newasian" = "Asian",

"race_cat_newhispanic_latino" = "Hispanic/Latinx" ,

"race_cat_newhawaiian_or_islander" = "Hawaiian/Islander" ,

"race_cat_newnative" = "American Indian/Alaska Native" ,

"race_cat_newmultiracial" = "Multiracial",

"race_cat_newother" = " Other",

"before_5_am" = "Before 5 a.m.",

"after_5_am" = "After 5 a.m.",

"regionsMidwest" = "Midwest",

"regionsSouth" = "South",

"regionsWest" = "West",

"month120-05_6" = "May-June, 2020",

#"month20-06" = "Jun, 2020",

"month120-07" = "Jul, 2020",

"month120-08" = "Aug, 2020",

"month120-09" = "Sep, 2020",

"month120-10" = "Oct, 2020",

"month120-11" = "Nov, 2020",

"month120-12" = "Dec, 2020",

"month121-01" = "Jan, 2021",

"month121-02" = "Feb, 2021",

"sleepless_than_5" = "less than 5",

"sleep5_to_6_hours" = "5 to 6 hrs",

"sleep9_to_10_hours" = "9 to 10 hrs",

"sleep11_or_more_hours" = "11 or more",

"live_aloneTRUE" = "Live Alone",

"income_cat[0,4.49e+04)" = "0-44.9k",

"income_cat[4.49e+04,6.57e+04)" = "44.9k-65.7k",

"income_cat[6.57e+04,Inf)" = "65.7k+",

"density_cat[150,1e+03)" = "150-1000",

"density_cat[1e+03,Inf)" = "1000+",

"feelingnot_well" = "Feeling unwell",

"weekendTRUE" = "Weekend",

"community_exposedTRUE" = "Community exposed",

"household_exposedTRUE" = "Household exposed",

"Case_rate" = "Case rate",

"Death_rate" = "Death rate",

"rt_greater_than_1TRUE" = "Rt > 1",

"covid_testedTRUE" = "Tested for COVID-19",

"covid_positiveTRUE" = "Positive for COVID-19",

"household_children_count1" = "1",

"household_children_count2" = "2",

"household_children_count3" = "3",

"household_children_count4" = "4",

"household_children_count5" = "5+",

"profession_essential_cathealthcare" = "Healthcare professional",

"profession_essential_catother_essential" = "Other essential workers",

"number_preexisting_cat1" = " 1",

"number_preexisting_cat2" = " 2",

"number_preexisting_cat3"= " 3",

"number_preexisting_cat4+" = " 4+"))

fit_grateful <- broom::tidy(geefit_grateful_ipw,conf.int = TRUE) %>% mutate(model = "Grateful") %>%

relabel_predictors(c("genderfemale" = "Female",

"genderother" = "Other",

"age_range_NEW[30,45)" = "30-45",

"age_range_NEW[45,60)" = "45-60",

"age_range_NEW[60,80)" = "60-80",

"age_range_NEW[80+)" = "80+",

"race_cat_newafrican_american" = "African American",

"race_cat_newasian" = "Asian",

"race_cat_newhispanic_latino" = "Hispanic/Latinx" ,

"race_cat_newhawaiian_or_islander" = "Hawaiian/Islander" ,

"race_cat_newnative" = "American Indian/Alaska Native" ,

"race_cat_newmultiracial" = "Multiracial",

"race_cat_newother" = " Other",

"before_5_am" = "Before 5 a.m.",

"after_5_am" = "After 5 a.m.",

"regionsMidwest" = "Midwest",

"regionsSouth" = "South",

"regionsWest" = "West",

"month120-05_6" = "May-June, 2020",

#"month20-06" = "Jun, 2020",

"month120-07" = "Jul, 2020",

"month120-08" = "Aug, 2020",

"month120-09" = "Sep, 2020",

"month120-10" = "Oct, 2020",

"month120-11" = "Nov, 2020",

"month120-12" = "Dec, 2020",

"month121-01" = "Jan, 2021",

"month121-02" = "Feb, 2021",

"sleepless_than_5" = "less than 5",

"sleep5_to_6_hours" = "5 to 6 hrs",

"sleep9_to_10_hours" = "9 to 10 hrs",

"sleep11_or_more_hours" = "11 or more",

"live_aloneTRUE" = "Live Alone",

"income_cat[0,4.49e+04)" = "0-44.9k",

"income_cat[4.49e+04,6.57e+04)" = "44.9k-65.7k",

"income_cat[6.57e+04,Inf)" = "65.7k+",

"density_cat[150,1e+03)" = "150-1000",

"density_cat[1e+03,Inf)" = "1000+",

"feelingnot_well" = "Feeling unwell",

"weekendTRUE" = "Weekend",

"community_exposedTRUE" = "Community exposed",

"household_exposedTRUE" = "Household exposed",

"Case_rate" = "Case rate",

"Death_rate" = "Death rate",

"rt_greater_than_1TRUE" = "Rt > 1",

"covid_testedTRUE" = "Tested for COVID-19",

"covid_positiveTRUE" = "Positive for COVID-19",

"household_children_count1" = "1",

"household_children_count2" = "2",

"household_children_count3" = "3",

"household_children_count4" = "4",

"household_children_count5" = "5+",

"profession_essential_cathealthcare" = "Healthcare professional",

"profession_essential_catother_essential" = "Other essential workers",

"number_preexisting_cat1" = " 1",

"number_preexisting_cat2" = " 2",

"number_preexisting_cat3"= " 3",

"number_preexisting_cat4+" = " 4+"))

fit_hopeful <- broom::tidy(geefit_hopeful_ipw,conf.int = TRUE) %>% mutate(model = "Hopeful") %>%

relabel_predictors(c("genderfemale" = "Female",

"genderother" = "Other",

"age_range_NEW[30,45)" = "30-45",

"age_range_NEW[45,60)" = "45-60",

"age_range_NEW[60,80)" = "60-80",

"age_range_NEW[80+)" = "80+",

"race_cat_newafrican_american" = "African American",

"race_cat_newasian" = "Asian",

"race_cat_newhispanic_latino" = "Hispanic/Latinx" ,

"race_cat_newhawaiian_or_islander" = "Hawaiian/Islander" ,

"race_cat_newnative" = "American Indian/Alaska Native" ,

"race_cat_newmultiracial" = "Multiracial",

"race_cat_newother" = " Other",

"before_5_am" = "Before 5 a.m.",

"after_5_am" = "After 5 a.m.",

"regionsMidwest" = "Midwest",

"regionsSouth" = "South",

"regionsWest" = "West",

"month120-05_6" = "May-June, 2020",

#"month20-06" = "Jun, 2020",

"month120-07" = "Jul, 2020",

"month120-08" = "Aug, 2020",

"month120-09" = "Sep, 2020",

"month120-10" = "Oct, 2020",

"month120-11" = "Nov, 2020",

"month120-12" = "Dec, 2020",

"month121-01" = "Jan, 2021",

"month121-02" = "Feb, 2021",

"sleepless_than_5" = "less than 5",

"sleep5_to_6_hours" = "5 to 6 hrs",

"sleep9_to_10_hours" = "9 to 10 hrs",

"sleep11_or_more_hours" = "11 or more",

"live_aloneTRUE" = "Live Alone",

"income_cat[0,4.49e+04)" = "0-44.9k",

"income_cat[4.49e+04,6.57e+04)" = "44.9k-65.7k",

"income_cat[6.57e+04,Inf)" = "65.7k+",

"density_cat[150,1e+03)" = "150-1000",

"density_cat[1e+03,Inf)" = "1000+",

"feelingnot_well" = "Feeling unwell",

"weekendTRUE" = "Weekend",

"community_exposedTRUE" = "Community exposed",

"household_exposedTRUE" = "Household exposed",

"Case_rate" = "Case rate",

"Death_rate" = "Death rate",

"rt_greater_than_1TRUE" = "Rt > 1",

"covid_testedTRUE" = "Tested for COVID-19",

"covid_positiveTRUE" = "Positive for COVID-19",

"household_children_count1" = "1",

"household_children_count2" = "2",

"household_children_count3" = "3",

"household_children_count4" = "4",

"household_children_count5" = "5+",

"profession_essential_cathealthcare" = "Healthcare professional",

"profession_essential_catother_essential" = "Other essential workers",

"number_preexisting_cat1" = " 1",

"number_preexisting_cat2" = " 2",

"number_preexisting_cat3"= " 3",

"number_preexisting_cat4+" = " 4+"))

fit_optimistic <- broom::tidy(geefit_optimistic_ipw,conf.int = TRUE) %>% mutate(model = "Optimistic") %>%

relabel_predictors(c("genderfemale" = "Female",

"genderother" = "Other",

"age_range_NEW[30,45)" = "30-45",

"age_range_NEW[45,60)" = "45-60",

"age_range_NEW[60,80)" = "60-80",

"age_range_NEW[80+)" = "80+",

"race_cat_newafrican_american" = "African American",

"race_cat_newasian" = "Asian",

"race_cat_newhispanic_latino" = "Hispanic/Latinx" ,

"race_cat_newhawaiian_or_islander" = "Hawaiian/Islander" ,

"race_cat_newnative" = "American Indian/Alaska Native" ,

"race_cat_newmultiracial" = "Multiracial",

"race_cat_newother" = " Other",

"before_5_am" = "Before 5 a.m.",

"after_5_am" = "After 5 a.m.",

"regionsMidwest" = "Midwest",

"regionsSouth" = "South",

"regionsWest" = "West",

"month120-05_6" = "May-June, 2020",

#"month20-06" = "Jun, 2020",

"month120-07" = "Jul, 2020",

"month120-08" = "Aug, 2020",

"month120-09" = "Sep, 2020",

"month120-10" = "Oct, 2020",

"month120-11" = "Nov, 2020",

"month120-12" = "Dec, 2020",

"month121-01" = "Jan, 2021",

"month121-02" = "Feb, 2021",

"sleepless_than_5" = "less than 5",

"sleep5_to_6_hours" = "5 to 6 hrs",

"sleep9_to_10_hours" = "9 to 10 hrs",

"sleep11_or_more_hours" = "11 or more",

"live_aloneTRUE" = "Live Alone",

"income_cat[0,4.49e+04)" = "0-44.9k",

"income_cat[4.49e+04,6.57e+04)" = "44.9k-65.7k",

"income_cat[6.57e+04,Inf)" = "65.7k+",

"density_cat[150,1e+03)" = "150-1000",

"density_cat[1e+03,Inf)" = "1000+",

"feelingnot_well" = "Feeling unwell",

"weekendTRUE" = "Weekend",

"community_exposedTRUE" = "Community exposed",

"household_exposedTRUE" = "Household exposed",

"Case_rate" = "Case rate",

"Death_rate" = "Death rate",

"rt_greater_than_1TRUE" = "Rt > 1",

"covid_testedTRUE" = "Tested for COVID-19",

"covid_positiveTRUE" = "Positive for COVID-19",

"household_children_count1" = "1",

"household_children_count2" = "2",

"household_children_count3" = "3",

"household_children_count4" = "4",

"household_children_count5" = "5+",

"profession_essential_cathealthcare" = "Healthcare professional",

"profession_essential_catother_essential" = "Other essential workers",

"number_preexisting_cat1" = " 1",

"number_preexisting_cat2" = " 2",

"number_preexisting_cat3"= " 3",

"number_preexisting_cat4+" = " 4+"))

demo_ind1 <- c(1:13,16:18)

demo_ind2 <- c(31:35,45:51)

time_ind <- c(19:26)

stressor_ind <- c(29,36:51)

fit_demo1 <- rbind(fit_happy[demo_ind1,],fit_optimistic[demo_ind1,],fit_hopeful[demo_ind1,],fit_calm[demo_ind1,],fit_grateful[demo_ind1,],fit_thoughtful[demo_ind1,])

{dwplot(fit_demo1, vline = geom_vline(xintercept = 0, colour = "grey60", linetype = 2),dot_args = list(size = 2.5), whisker_args = list(size = 1))+theme_bw()+theme(plot.title = element_text(face="bold"), text=element_text(size=25, face="bold"))+ scale_color_manual(values = c( "#00468BFF", "black", "#42B540FF", "#0099B4FF", "#925E9FFF", "#FDAF91FF"))}%>%

add_brackets(brackets_demo1, fontSize = 1.5, face="bold")

ggsave('/ncf/xlin_covid/Users/sshen/dat5_02_09/HWF_plots/pos_demo1.pdf',height = 15,width = 15)

fit_demo2 <- rbind(fit_happy[demo_ind2,],fit_optimistic[demo_ind2,],fit_hopeful[demo_ind2,],fit_calm[demo_ind2,],fit_grateful[demo_ind2,],fit_thoughtful[demo_ind2,])

{dwplot(fit_demo2, vline = geom_vline(xintercept = 0, colour = "grey60", linetype = 2), dot_args = list(size = 2.5), whisker_args = list(size = 1))+theme_bw()+theme(plot.title = element_text(face="bold"), text=element_text(size=25, face="bold"))+ scale_color_manual(values = c( "#00468BFF", "black", "#42B540FF", "#0099B4FF", "#925E9FFF", "#FDAF91FF"))}%>%

add_brackets(brackets_demo2, fontSize = 1.5, face="bold")

ggsave('/ncf/xlin_covid/Users/sshen/dat5_02_09/HWF_plots/pos_demo2.pdf',height = 15,width = 15)

fit_time <- rbind(fit_happy[time_ind,],fit_optimistic[time_ind,],fit_hopeful[time_ind,],fit_calm[time_ind,],fit_grateful[time_ind,],fit_thoughtful[time_ind,])

{dwplot(fit_time, vline = geom_vline(xintercept = 0, colour = "grey60", linetype = 2), dot_args = list(size = 2.5), whisker_args = list(size = 1))+theme_bw()+theme(plot.title = element_text(face="bold"), text=element_text(size=18, face="bold"))+ scale_color_manual(values = c( "#00468BFF", "black", "#42B540FF", "#0099B4FF", "#925E9FFF", "#FDAF91FF"))}%>%

add_brackets(brackets_time, fontSize = 1.2, face="bold")

ggsave('/ncf/xlin_covid/Users/sshen/dat5_02_09/HWF_plots/pos_time.pdf',height = 8,width = 10)

save(fit_demo1, fit_demo2,fit_time,file = "/ncf/xlin_covid/Users/sshen/dat5_02_09/results/fit_demo_time_pos.RData")

#{dwplot(rbind(fit_happy[stressor_ind,],fit_optimistic[stressor_ind,],fit_hopeful[stressor_ind,],fit_calm[stressor_ind,],fit_grateful[stressor_ind,],fit_thoughtful[stressor_ind,]), vline = geom_vline(xintercept = 0, colour = "grey60", linetype = 2))+theme_bw()+theme(plot.title = element_text(face="bold"))+ scale_color_manual(values = c( "#FC8D62", "#8DA0CB", "#E78AC3", "#A6D854", "#FFD92F", "#E5C494", "#B3B3B3"))}%>%

# add_brackets(brackets_stressor)

#ggsave('/ncf/xlin_covid/Users/sshen/dat5_02_09/HWF_plots/pos_stressor.png',height = 10,width = 8)

reg_protect_behav_neg.R

library(geepack)

library(dotwhisker)

library(dplyr)

library(ggsci)

library(RColorBrewer)

load('/ncf/xlin_covid/Users/sshen/dat5_02_09/dat_prot.RData')

dat_ipw_complete <- dat_ipw_complete[!is.na(dat_ipw_complete$stay_home_1w_5), ]

#dat_prot1 <- dat_prot1[!is.na(dat_prot1$social_dist_1w_5), ]

#angry, lonely, sad, stressed, anxious, tired

#angry, lonely, sad, stressed, anxious, tired

####stayhome

geefit_angry_ipw<-geeglm(emotions_angry~age_range_NEW+ gender+race_cat+ before_5_am+after_5_am+regions+ month1+sleep+live_alone+income_cat+density_cat+ feeling+weekend+community_exposed + household_exposed +rt_greater_than_1+covid_tested+covid_positive+child_count_cat+profession_essential_cat+number_preexisting_cat+stay_home_1w_75, id=session_id, family=gaussian(), corstr="independence", data=dat_ipw_complete,weights=weights)

geefit_lonely_ipw<-geeglm(emotions_lonely~age_range_NEW+ gender+race_cat+ before_5_am+after_5_am+regions+ month1+sleep+live_alone+income_cat+density_cat+ feeling+weekend+community_exposed + household_exposed +rt_greater_than_1+covid_tested+covid_positive+child_count_cat+profession_essential_cat+number_preexisting_cat+stay_home_1w_75, id=session_id, family=gaussian(), corstr="independence", data=dat_ipw_complete,weights=weights)

geefit_sad_ipw<-geeglm(emotions_sad~age_range_NEW+ gender+race_cat+ before_5_am+after_5_am+regions+ month1+sleep+live_alone+income_cat+density_cat+ feeling+weekend+community_exposed + household_exposed +rt_greater_than_1+covid_tested+covid_positive+child_count_cat+profession_essential_cat+number_preexisting_cat+stay_home_1w_75, id=session_id, family=gaussian(), corstr="independence", data=dat_ipw_complete,weights=weights)

geefit_stressed_ipw<-geeglm(emotions_stressed~age_range_NEW+ gender+race_cat+ before_5_am+after_5_am+regions+ month1+sleep+live_alone+income_cat+density_cat+ feeling+weekend+community_exposed + household_exposed +rt_greater_than_1+covid_tested+covid_positive+child_count_cat+profession_essential_cat+number_preexisting_cat+stay_home_1w_75, id=session_id, family=gaussian(), corstr="independence", data=dat_ipw_complete,weights=weights)

geefit_anxious_ipw<-geeglm(emotions_anxious~age_range_NEW+ gender+race_cat+ before_5_am+after_5_am+regions+ month1+sleep+live_alone+income_cat+density_cat+ feeling+weekend+community_exposed + household_exposed +rt_greater_than_1+covid_tested+covid_positive+child_count_cat+profession_essential_cat+number_preexisting_cat+stay_home_1w_75, id=session_id, family=gaussian(), corstr="independence", data=dat_ipw_complete,weights=weights)

geefit_tired_ipw<-geeglm(emotions_tired~age_range_NEW+ gender+race_cat+ before_5_am+after_5_am+regions+ month1+sleep+live_alone+income_cat+density_cat+ feeling+weekend+community_exposed + household_exposed +rt_greater_than_1+covid_tested+covid_positive+child_count_cat+profession_essential_cat+number_preexisting_cat+stay_home_1w_75, id=session_id, family=gaussian(), corstr="independence", data=dat_ipw_complete,weights=weights)

fit_angry1 <- broom::tidy(geefit_angry_ipw,conf.int = TRUE) %>% mutate(model = "Angry") %>%

relabel_predictors(c(

"stay_home_1w_75TRUE" = "Stayed home"))

fit_lonely1 <- broom::tidy(geefit_lonely_ipw,conf.int = TRUE) %>% mutate(model = "Lonely") %>%

relabel_predictors(c(

"stay_home_1w_75TRUE" = "Stayed home"))

fit_sad1 <- broom::tidy(geefit_sad_ipw,conf.int = TRUE) %>% mutate(model = "Sad") %>%

relabel_predictors(c(

"stay_home_1w_75TRUE" = "Stayed home"))

fit_stressed1 <- broom::tidy(geefit_stressed_ipw,conf.int = TRUE) %>% mutate(model = "Stressed") %>%

relabel_predictors(c(

"stay_home_1w_75TRUE" = "Stayed home"))

fit_anxious1 <- broom::tidy(geefit_anxious_ipw,conf.int = TRUE) %>% mutate(model = "Anxious") %>%

relabel_predictors(c(

"stay_home_1w_75TRUE" = "Stayed home"))

fit_tired1 <- broom::tidy(geefit_tired_ipw,conf.int = TRUE) %>% mutate(model = "Tired") %>%

relabel_predictors(c(

"stay_home_1w_75TRUE" = "Stayed home"))

####face cover

geefit_angry_ipw<-geeglm(emotions_angry~age_range_NEW+ gender+race_cat+ before_5_am+after_5_am+regions+ month1+sleep+live_alone+income_cat+density_cat+ feeling+weekend+community_exposed + household_exposed +rt_greater_than_1+covid_tested+covid_positive+child_count_cat+profession_essential_cat+number_preexisting_cat+face_covering_1w_75, id=session_id, family=gaussian(), corstr="independence", data=dat_prot2,weights=weights)

geefit_lonely_ipw<-geeglm(emotions_lonely~age_range_NEW+ gender+race_cat+ before_5_am+after_5_am+regions+ month1+sleep+live_alone+income_cat+density_cat+ feeling+weekend+community_exposed + household_exposed +rt_greater_than_1+covid_tested+covid_positive+child_count_cat+profession_essential_cat+number_preexisting_cat+face_covering_1w_75, id=session_id, family=gaussian(), corstr="independence", data=dat_prot2,weights=weights)

geefit_sad_ipw<-geeglm(emotions_sad~age_range_NEW+ gender+race_cat+ before_5_am+after_5_am+regions+ month1+sleep+live_alone+income_cat+density_cat+ feeling+weekend+community_exposed + household_exposed +rt_greater_than_1+covid_tested+covid_positive+child_count_cat+profession_essential_cat+number_preexisting_cat+face_covering_1w_75, id=session_id, family=gaussian(), corstr="independence", data=dat_prot2,weights=weights)

geefit_stressed_ipw<-geeglm(emotions_stressed~age_range_NEW+ gender+race_cat+ before_5_am+after_5_am+regions+ month1+sleep+live_alone+income_cat+density_cat+ feeling+weekend+community_exposed + household_exposed +rt_greater_than_1+covid_tested+covid_positive+child_count_cat+profession_essential_cat+number_preexisting_cat+face_covering_1w_75, id=session_id, family=gaussian(), corstr="independence", data=dat_prot2,weights=weights)

geefit_anxious_ipw<-geeglm(emotions_anxious~age_range_NEW+ gender+race_cat+ before_5_am+after_5_am+regions+ month1+sleep+live_alone+income_cat+density_cat+ feeling+weekend+community_exposed + household_exposed +rt_greater_than_1+covid_tested+covid_positive+child_count_cat+profession_essential_cat+number_preexisting_cat+face_covering_1w_75, id=session_id, family=gaussian(), corstr="independence", data=dat_prot2,weights=weights)

geefit_tired_ipw<-geeglm(emotions_tired~age_range_NEW+ gender+race_cat+ before_5_am+after_5_am+regions+ month1+sleep+live_alone+income_cat+density_cat+ feeling+weekend+community_exposed + household_exposed +rt_greater_than_1+covid_tested+covid_positive+child_count_cat+profession_essential_cat+number_preexisting_cat+face_covering_1w_75, id=session_id, family=gaussian(), corstr="independence", data=dat_prot2,weights=weights)

fit_angry2 <- broom::tidy(geefit_angry_ipw,conf.int = TRUE) %>% mutate(model = "Angry") %>%

relabel_predictors(c(

"face_covering_1w_75TRUE" = "Face covering"))

fit_lonely2 <- broom::tidy(geefit_lonely_ipw,conf.int = TRUE) %>% mutate(model = "Lonely") %>%

relabel_predictors(c(

"face_covering_1w_75TRUE" = "Face covering"))

fit_sad2 <- broom::tidy(geefit_sad_ipw,conf.int = TRUE) %>% mutate(model = "Sad") %>%

relabel_predictors(c(

"face_covering_1w_75TRUE" = "Face covering"))

fit_stressed2 <- broom::tidy(geefit_stressed_ipw,conf.int = TRUE) %>% mutate(model = "Stressed") %>%

relabel_predictors(c(

"face_covering_1w_75TRUE" = "Face covering"))

fit_anxious2 <- broom::tidy(geefit_anxious_ipw,conf.int = TRUE) %>% mutate(model = "Anxious") %>%

relabel_predictors(c(

"face_covering_1w_75TRUE" = "Face covering"))

fit_tired2 <- broom::tidy(geefit_tired_ipw,conf.int = TRUE) %>% mutate(model = "Tired") %>%

relabel_predictors(c(

"face_covering_1w_75TRUE" = "Face covering"))

####social dist

geefit_angry_ipw<-geeglm(emotions_angry~age_range_NEW+ gender+race_cat+ before_5_am+after_5_am+regions+ month1+sleep+live_alone+income_cat+density_cat+ feeling+weekend+community_exposed + household_exposed +rt_greater_than_1+covid_tested+covid_positive+child_count_cat+profession_essential_cat+number_preexisting_cat+social_dist_1w_75, id=session_id, family=gaussian(), corstr="independence", data=dat_prot1,weights=weights)

geefit_lonely_ipw<-geeglm(emotions_lonely~age_range_NEW+ gender+race_cat+ before_5_am+after_5_am+regions+ month1+sleep+live_alone+income_cat+density_cat+ feeling+weekend+community_exposed + household_exposed +rt_greater_than_1+covid_tested+covid_positive+child_count_cat+profession_essential_cat+number_preexisting_cat+social_dist_1w_75, id=session_id, family=gaussian(), corstr="independence", data=dat_prot1,weights=weights)

geefit_sad_ipw<-geeglm(emotions_sad~age_range_NEW+ gender+race_cat+ before_5_am+after_5_am+regions+ month1+sleep+live_alone+income_cat+density_cat+ feeling+weekend+community_exposed + household_exposed +rt_greater_than_1+covid_tested+covid_positive+child_count_cat+profession_essential_cat+number_preexisting_cat+social_dist_1w_75, id=session_id, family=gaussian(), corstr="independence", data=dat_prot1,weights=weights)

geefit_stressed_ipw<-geeglm(emotions_stressed~age_range_NEW+ gender+race_cat+ before_5_am+after_5_am+regions+ month1+sleep+live_alone+income_cat+density_cat+ feeling+weekend+community_exposed + household_exposed +rt_greater_than_1+covid_tested+covid_positive+child_count_cat+profession_essential_cat+number_preexisting_cat+social_dist_1w_75, id=session_id, family=gaussian(), corstr="independence", data=dat_prot1,weights=weights)

geefit_anxious_ipw<-geeglm(emotions_anxious~age_range_NEW+ gender+race_cat+ before_5_am+after_5_am+regions+ month1+sleep+live_alone+income_cat+density_cat+ feeling+weekend+community_exposed + household_exposed +rt_greater_than_1+covid_tested+covid_positive+child_count_cat+profession_essential_cat+number_preexisting_cat+social_dist_1w_75, id=session_id, family=gaussian(), corstr="independence", data=dat_prot1,weights=weights)

geefit_tired_ipw<-geeglm(emotions_tired~age_range_NEW+ gender+race_cat+ before_5_am+after_5_am+regions+ month1+sleep+live_alone+income_cat+density_cat+ feeling+weekend+community_exposed + household_exposed +rt_greater_than_1+covid_tested+covid_positive+child_count_cat+profession_essential_cat+number_preexisting_cat+social_dist_1w_75, id=session_id, family=gaussian(), corstr="independence", data=dat_prot1,weights=weights)

fit_angry3 <- broom::tidy(geefit_angry_ipw,conf.int = TRUE) %>% mutate(model = "Angry") %>%

relabel_predictors(c(

"social_dist_1w_75TRUE" = "Social distancing"))

fit_lonely3 <- broom::tidy(geefit_lonely_ipw,conf.int = TRUE) %>% mutate(model = "Lonely") %>%

relabel_predictors(c(

"social_dist_1w_75TRUE" = "Social distancing"))

fit_sad3 <- broom::tidy(geefit_sad_ipw,conf.int = TRUE) %>% mutate(model = "Sad") %>%

relabel_predictors(c(

"social_dist_1w_75TRUE" = "Social distancing"))

fit_stressed3 <- broom::tidy(geefit_stressed_ipw,conf.int = TRUE) %>% mutate(model = "Stressed") %>%

relabel_predictors(c(

"social_dist_1w_75TRUE" = "Social distancing"))

fit_anxious3 <- broom::tidy(geefit_anxious_ipw,conf.int = TRUE) %>% mutate(model = "Anxious") %>%

relabel_predictors(c(

"social_dist_1w_75TRUE" = "Social distancing"))

fit_tired3 <- broom::tidy(geefit_tired_ipw,conf.int = TRUE) %>% mutate(model = "Tired") %>%

relabel_predictors(c(

"social_dist_1w_75TRUE" = "Social distancing"))

fit_comb <- rbind(fit_angry1[1,],

fit_lonely1[1,],

fit_sad1[1,],

fit_stressed1[1,],

fit_anxious1[1,],

fit_tired1[1,],

fit_angry2[1,],

fit_lonely2[1,],

fit_sad2[1,],

fit_stressed2[1,],

fit_anxious2[1,],

fit_tired2[1,],

fit_angry3[1,],

fit_lonely3[1,],

fit_sad3[1,],

fit_stressed3[1,],

fit_anxious3[1,],

fit_tired3[1,]

)

{dwplot(fit_comb, vline = geom_vline(xintercept = 0, colour = "grey60", linetype = 2),dot_args = list(size = 2.5), whisker_args = list(size = 1)) +theme_bw()+theme(plot.title = element_text(face="bold"), text=element_text(size=17, face = 'bold'))}+scale_color_manual(values = c( "#00468BFF", "black", "#42B540FF", "#0099B4FF", "#925E9FFF", "#FDAF91FF"))

ggsave('/ncf/xlin_covid/Users/sshen/dat5_02_09/HWF_plots/prot_behav_neg.pdf',height = 6,width = 8)

save(fit_comb,file = "/ncf/xlin_covid/Users/sshen/dat5_02_09/results/fit_prot_neg.RData")

library(geepack)

library(dotwhisker)

library(dplyr)

library(ggsci)

library(RColorBrewer)

load('/ncf/xlin_covid/Users/sshen/dat5_02_09/dat_prot.RData')

dat_ipw_complete <- dat_ipw_complete[!is.na(dat_ipw_complete$stay_home_1w_5), ]

#dat_prot1 <- dat_prot1[!is.na(dat_prot1$social_dist_1w_5), ]

#happy, optimistic, hopeful, calm, grateful, thoughtful

#angry, lonely, sad, stressed, anxious, tired

####stayhome

geefit_happy_ipw<-geeglm(emotions_happy~age_range_NEW+ gender+race_cat+ before_5_am+after_5_am+regions+ month1+sleep+live_alone+income_cat+density_cat+ feeling+weekend+community_exposed + household_exposed +rt_greater_than_1+covid_tested+covid_positive+child_count_cat+profession_essential_cat+number_preexisting_cat+stay_home_1w_75, id=session_id, family=gaussian(), corstr="independence", data=dat_ipw_complete,weights=weights)

geefit_optimistic_ipw<-geeglm(emotions_optimistic~age_range_NEW+ gender+race_cat+ before_5_am+after_5_am+regions+ month1+sleep+live_alone+income_cat+density_cat+ feeling+weekend+community_exposed + household_exposed +rt_greater_than_1+covid_tested+covid_positive+child_count_cat+profession_essential_cat+number_preexisting_cat+stay_home_1w_75, id=session_id, family=gaussian(), corstr="independence", data=dat_ipw_complete,weights=weights)

geefit_hopeful_ipw<-geeglm(emotions_hopeful~age_range_NEW+ gender+race_cat+ before_5_am+after_5_am+regions+ month1+sleep+live_alone+income_cat+density_cat+ feeling+weekend+community_exposed + household_exposed +rt_greater_than_1+covid_tested+covid_positive+child_count_cat+profession_essential_cat+number_preexisting_cat+stay_home_1w_75, id=session_id, family=gaussian(), corstr="independence", data=dat_ipw_complete,weights=weights)

geefit_calm_ipw<-geeglm(emotions_calm~age_range_NEW+ gender+race_cat+ before_5_am+after_5_am+regions+ month1+sleep+live_alone+income_cat+density_cat+ feeling+weekend+community_exposed + household_exposed +rt_greater_than_1+covid_tested+covid_positive+child_count_cat+profession_essential_cat+number_preexisting_cat+stay_home_1w_75, id=session_id, family=gaussian(), corstr="independence", data=dat_ipw_complete,weights=weights)

geefit_grateful_ipw<-geeglm(emotions_grateful~age_range_NEW+ gender+race_cat+ before_5_am+after_5_am+regions+ month1+sleep+live_alone+income_cat+density_cat+ feeling+weekend+community_exposed + household_exposed +rt_greater_than_1+covid_tested+covid_positive+child_count_cat+profession_essential_cat+number_preexisting_cat+stay_home_1w_75, id=session_id, family=gaussian(), corstr="independence", data=dat_ipw_complete,weights=weights)

geefit_thoughtful_ipw<-geeglm(emotions_thoughtful~age_range_NEW+ gender+race_cat+ before_5_am+after_5_am+regions+ month1+sleep+live_alone+income_cat+density_cat+ feeling+weekend+community_exposed + household_exposed +rt_greater_than_1+covid_tested+covid_positive+child_count_cat+profession_essential_cat+number_preexisting_cat+stay_home_1w_75, id=session_id, family=gaussian(), corstr="independence", data=dat_ipw_complete,weights=weights)

fit_happy1 <- broom::tidy(geefit_happy_ipw,conf.int = TRUE) %>% mutate(model = "Happy") %>%

relabel_predictors(c(

"stay_home_1w_75TRUE" = "Stayed home"))

fit_optimistic1 <- broom::tidy(geefit_optimistic_ipw,conf.int = TRUE) %>% mutate(model = "Optimistic") %>%

relabel_predictors(c(

"stay_home_1w_75TRUE" = "Stayed home"))

fit_hopeful1 <- broom::tidy(geefit_hopeful_ipw,conf.int = TRUE) %>% mutate(model = "Hopeful") %>%

relabel_predictors(c(

"stay_home_1w_75TRUE" = "Stayed home"))

fit_calm1 <- broom::tidy(geefit_calm_ipw,conf.int = TRUE) %>% mutate(model = "Calm") %>%

relabel_predictors(c(

"stay_home_1w_75TRUE" = "Stayed home"))

fit_grateful1 <- broom::tidy(geefit_grateful_ipw,conf.int = TRUE) %>% mutate(model = "Grateful") %>%

relabel_predictors(c(

"stay_home_1w_75TRUE" = "Stayed home"))

fit_thoughtful1 <- broom::tidy(geefit_thoughtful_ipw,conf.int = TRUE) %>% mutate(model = "Thoughtful") %>%

relabel_predictors(c(

"stay_home_1w_75TRUE" = "Stayed home"))

####face cover

geefit_happy_ipw<-geeglm(emotions_happy~age_range_NEW+ gender+race_cat+ before_5_am+after_5_am+regions+ month1+sleep+live_alone+income_cat+density_cat+ feeling+weekend+community_exposed + household_exposed +rt_greater_than_1+covid_tested+covid_positive+child_count_cat+profession_essential_cat+number_preexisting_cat+face_covering_1w_75, id=session_id, family=gaussian(), corstr="independence", data=dat_prot2,weights=weights)

geefit_optimistic_ipw<-geeglm(emotions_optimistic~age_range_NEW+ gender+race_cat+ before_5_am+after_5_am+regions+ month1+sleep+live_alone+income_cat+density_cat+ feeling+weekend+community_exposed + household_exposed +rt_greater_than_1+covid_tested+covid_positive+child_count_cat+profession_essential_cat+number_preexisting_cat+face_covering_1w_75, id=session_id, family=gaussian(), corstr="independence", data=dat_prot2,weights=weights)

geefit_hopeful_ipw<-geeglm(emotions_hopeful~age_range_NEW+ gender+race_cat+ before_5_am+after_5_am+regions+ month1+sleep+live_alone+income_cat+density_cat+ feeling+weekend+community_exposed + household_exposed +rt_greater_than_1+covid_tested+covid_positive+child_count_cat+profession_essential_cat+number_preexisting_cat+face_covering_1w_75, id=session_id, family=gaussian(), corstr="independence", data=dat_prot2,weights=weights)

geefit_calm_ipw<-geeglm(emotions_calm~age_range_NEW+ gender+race_cat+ before_5_am+after_5_am+regions+ month1+sleep+live_alone+income_cat+density_cat+ feeling+weekend+community_exposed + household_exposed +rt_greater_than_1+covid_tested+covid_positive+child_count_cat+profession_essential_cat+number_preexisting_cat+face_covering_1w_75, id=session_id, family=gaussian(), corstr="independence", data=dat_prot2,weights=weights)

geefit_grateful_ipw<-geeglm(emotions_grateful~age_range_NEW+ gender+race_cat+ before_5_am+after_5_am+regions+ month1+sleep+live_alone+income_cat+density_cat+ feeling+weekend+community_exposed + household_exposed +rt_greater_than_1+covid_tested+covid_positive+child_count_cat+profession_essential_cat+number_preexisting_cat+face_covering_1w_75, id=session_id, family=gaussian(), corstr="independence", data=dat_prot2,weights=weights)

geefit_thoughtful_ipw<-geeglm(emotions_thoughtful~age_range_NEW+ gender+race_cat+ before_5_am+after_5_am+regions+ month1+sleep+live_alone+income_cat+density_cat+ feeling+weekend+community_exposed + household_exposed +rt_greater_than_1+covid_tested+covid_positive+child_count_cat+profession_essential_cat+number_preexisting_cat+face_covering_1w_75, id=session_id, family=gaussian(), corstr="independence", data=dat_prot2,weights=weights)

fit_happy2 <- broom::tidy(geefit_happy_ipw,conf.int = TRUE) %>% mutate(model = "Happy") %>%

relabel_predictors(c(

"face_covering_1w_75TRUE" = "Face covering"))

fit_optimistic2 <- broom::tidy(geefit_optimistic_ipw,conf.int = TRUE) %>% mutate(model = "Optimistic") %>%

relabel_predictors(c(

"face_covering_1w_75TRUE" = "Face covering"))

fit_hopeful2 <- broom::tidy(geefit_hopeful_ipw,conf.int = TRUE) %>% mutate(model = "Hopeful") %>%

relabel_predictors(c(

"face_covering_1w_75TRUE" = "Face covering"))

fit_calm2 <- broom::tidy(geefit_calm_ipw,conf.int = TRUE) %>% mutate(model = "Calm") %>%

relabel_predictors(c(

"face_covering_1w_75TRUE" = "Face covering"))

fit_grateful2 <- broom::tidy(geefit_grateful_ipw,conf.int = TRUE) %>% mutate(model = "Grateful") %>%

relabel_predictors(c(

"face_covering_1w_75TRUE" = "Face covering"))

fit_thoughtful2 <- broom::tidy(geefit_thoughtful_ipw,conf.int = TRUE) %>% mutate(model = "Thoughtful") %>%

relabel_predictors(c(

"face_covering_1w_75TRUE" = "Face covering"))

####social dist

geefit_happy_ipw<-geeglm(emotions_happy~age_range_NEW+ gender+race_cat+ before_5_am+after_5_am+regions+ month1+sleep+live_alone+income_cat+density_cat+ feeling+weekend+community_exposed + household_exposed +rt_greater_than_1+covid_tested+covid_positive+child_count_cat+profession_essential_cat+number_preexisting_cat+social_dist_1w_75, id=session_id, family=gaussian(), corstr="independence", data=dat_prot1,weights=weights)

geefit_optimistic_ipw<-geeglm(emotions_optimistic~age_range_NEW+ gender+race_cat+ before_5_am+after_5_am+regions+ month1+sleep+live_alone+income_cat+density_cat+ feeling+weekend+community_exposed + household_exposed +rt_greater_than_1+covid_tested+covid_positive+child_count_cat+profession_essential_cat+number_preexisting_cat+social_dist_1w_75, id=session_id, family=gaussian(), corstr="independence", data=dat_prot1,weights=weights)

geefit_hopeful_ipw<-geeglm(emotions_hopeful~age_range_NEW+ gender+race_cat+ before_5_am+after_5_am+regions+ month1+sleep+live_alone+income_cat+density_cat+ feeling+weekend+community_exposed + household_exposed +rt_greater_than_1+covid_tested+covid_positive+child_count_cat+profession_essential_cat+number_preexisting_cat+social_dist_1w_75, id=session_id, family=gaussian(), corstr="independence", data=dat_prot1,weights=weights)

geefit_calm_ipw<-geeglm(emotions_calm~age_range_NEW+ gender+race_cat+ before_5_am+after_5_am+regions+ month1+sleep+live_alone+income_cat+density_cat+ feeling+weekend+community_exposed + household_exposed +rt_greater_than_1+covid_tested+covid_positive+child_count_cat+profession_essential_cat+number_preexisting_cat+social_dist_1w_75, id=session_id, family=gaussian(), corstr="independence", data=dat_prot1,weights=weights)

geefit_grateful_ipw<-geeglm(emotions_grateful~age_range_NEW+ gender+race_cat+ before_5_am+after_5_am+regions+ month1+sleep+live_alone+income_cat+density_cat+ feeling+weekend+community_exposed + household_exposed +rt_greater_than_1+covid_tested+covid_positive+child_count_cat+profession_essential_cat+number_preexisting_cat+social_dist_1w_75, id=session_id, family=gaussian(), corstr="independence", data=dat_prot1,weights=weights)

geefit_thoughtful_ipw<-geeglm(emotions_thoughtful~age_range_NEW+ gender+race_cat+ before_5_am+after_5_am+regions+ month1+sleep+live_alone+income_cat+density_cat+ feeling+weekend+community_exposed + household_exposed +rt_greater_than_1+covid_tested+covid_positive+child_count_cat+profession_essential_cat+number_preexisting_cat+social_dist_1w_75, id=session_id, family=gaussian(), corstr="independence", data=dat_prot1,weights=weights)

fit_happy3 <- broom::tidy(geefit_happy_ipw,conf.int = TRUE) %>% mutate(model = "Happy") %>%

relabel_predictors(c(

"social_dist_1w_75TRUE" = "Social distancing"))

fit_optimistic3 <- broom::tidy(geefit_optimistic_ipw,conf.int = TRUE) %>% mutate(model = "Optimistic") %>%

relabel_predictors(c(

"social_dist_1w_75TRUE" = "Social distancing"))

fit_hopeful3 <- broom::tidy(geefit_hopeful_ipw,conf.int = TRUE) %>% mutate(model = "Hopeful") %>%

relabel_predictors(c(

"social_dist_1w_75TRUE" = "Social distancing"))

fit_calm3 <- broom::tidy(geefit_calm_ipw,conf.int = TRUE) %>% mutate(model = "Calm") %>%

relabel_predictors(c(

"social_dist_1w_75TRUE" = "Social distancing"))

fit_grateful3 <- broom::tidy(geefit_grateful_ipw,conf.int = TRUE) %>% mutate(model = "Grateful") %>%

relabel_predictors(c(

"social_dist_1w_75TRUE" = "Social distancing"))

fit_thoughtful3 <- broom::tidy(geefit_thoughtful_ipw,conf.int = TRUE) %>% mutate(model = "Thoughtful") %>%

relabel_predictors(c(

"social_dist_1w_75TRUE" = "Social distancing"))

fit_comb <- rbind(fit_happy1[1,],

fit_optimistic1[1,],

fit_hopeful1[1,],

fit_calm1[1,],

fit_grateful1[1,],

fit_thoughtful1[1,],

fit_happy2[1,],

fit_optimistic2[1,],

fit_hopeful2[1,],

fit_calm2[1,],

fit_grateful2[1,],

fit_thoughtful2[1,],

fit_happy3[1,],

fit_optimistic3[1,],

fit_hopeful3[1,],

fit_calm3[1,],

fit_grateful3[1,],

fit_thoughtful3[1,]

)

{dwplot(fit_comb, vline = geom_vline(xintercept = 0, colour = "grey60", linetype = 2),dot_args = list(size = 2.5), whisker_args = list(size = 1))+theme_bw()+theme(plot.title = element_text(face="bold"), text=element_text(size=17, face = 'bold'))}+ scale_color_manual(values = c( "#00468BFF", "black", "#42B540FF", "#0099B4FF", "#925E9FFF", "#FDAF91FF"))

ggsave('/ncf/xlin_covid/Users/sshen/dat5_02_09/HWF_plots/prot_behav_pos.pdf',height = 6,width = 8)

save(fit_comb,file = "/ncf/xlin_covid/Users/sshen/dat5_02_09/results/fit_prot_pos.RData")

library(geepack)

library(dotwhisker)

library(dplyr)

library(ggsci)

library(RColorBrewer)

load('/ncf/xlin_covid/Users/sshen/dat5_02_09/reg_data1.RData')

dat_ipw_complete$income_cat <- relevel(dat_ipw_complete$income_cat, ref = "[0,4.49e+04)")

#angry, lonely, sad, stressed, anxious, tired

#angry, lonely, sad, stressed, anxious, tired

geefit_angry_ipw<-geeglm(emotions_angry~occup_score + family_score + number_preexisting + symp_score + demo_score + exposure_score + rt_greater_than_1 + gender + before_5_am+after_5_am+regions+ month1+income_cat+ feeling+weekend +Case_rate +Death_rate+covid_tested+covid_tested+covid_positive+sleep_score , id=session_id, family=gaussian(), corstr="independence", data=dat_ipw_complete,weights=weights)

geefit_lonely_ipw<-geeglm(emotions_lonely~occup_score + family_score + number_preexisting + symp_score + demo_score + exposure_score + rt_greater_than_1 + gender + before_5_am+after_5_am+regions+ month1+income_cat+ feeling+weekend +Case_rate +Death_rate+covid_tested+covid_tested+covid_positive+sleep_score , id=session_id, family=gaussian(), corstr="independence", data=dat_ipw_complete,weights=weights)

geefit_sad_ipw<-geeglm(emotions_sad~occup_score + family_score + number_preexisting + symp_score + demo_score + exposure_score + rt_greater_than_1 + gender + before_5_am+after_5_am+regions+ month1+income_cat+ feeling+weekend +Case_rate +Death_rate+covid_tested+covid_tested+covid_positive+sleep_score , id=session_id, family=gaussian(), corstr="independence", data=dat_ipw_complete,weights=weights)

geefit_stressed_ipw<-geeglm(emotions_stressed~occup_score + family_score + number_preexisting + symp_score + demo_score + exposure_score + rt_greater_than_1 + gender + before_5_am+after_5_am+regions+ month1+income_cat+ feeling+weekend +Case_rate +Death_rate+covid_tested+covid_tested+covid_positive+sleep_score , id=session_id, family=gaussian(), corstr="independence", data=dat_ipw_complete,weights=weights)

geefit_anxious_ipw<-geeglm(emotions_anxious~occup_score + family_score + number_preexisting + symp_score + demo_score + exposure_score + rt_greater_than_1 + gender + before_5_am+after_5_am+regions+ month1+income_cat+ feeling+weekend +Case_rate +Death_rate+covid_tested+covid_tested+covid_positive+sleep_score , id=session_id, family=gaussian(), corstr="independence", data=dat_ipw_complete,weights=weights)

geefit_tired_ipw<-geeglm(emotions_tired~occup_score + family_score + number_preexisting + symp_score + demo_score + exposure_score + rt_greater_than_1 + gender + before_5_am+after_5_am+regions+ month1+income_cat+ feeling+weekend +Case_rate +Death_rate+covid_tested+covid_tested+covid_positive+sleep_score , id=session_id, family=gaussian(), corstr="independence", data=dat_ipw_complete,weights=weights)

fit_angry <- broom::tidy(geefit_angry_ipw,conf.int = TRUE) %>% mutate(model = "Angry") %>%

relabel_predictors(c(

"rt_greater_than_1TRUE" = "Rt value",

"occup_score" = "Occupational stressors",

"family_score" = "Family stressors",

"number_preexisting" = "Health stressors",

"symp_score" = "Symptom stressors",

"demo_score" = "Demographic stressors",

"exposure_score" = "Exposure stressors",

"sleep_score" = "Sleep stressors",

"feelingnot_well" = "Feeling unwell"

))

fit_lonely <- broom::tidy(geefit_lonely_ipw,conf.int = TRUE) %>% mutate(model = "Lonely") %>%

relabel_predictors(c(

"rt_greater_than_1TRUE" = "Rt value",

"occup_score" = "Occupational stressors",

"family_score" = "Family stressors",

"number_preexisting" = "Health stressors",

"symp_score" = "Symptom stressors",

"demo_score" = "Demographic stressors",

"exposure_score" = "Exposure stressors",

"sleep_score" = "Sleep stressors",

"feelingnot_well" = "Feeling unwell"

))

fit_sad <- broom::tidy(geefit_sad_ipw,conf.int = TRUE) %>% mutate(model = "Sad") %>%

relabel_predictors(c(

"rt_greater_than_1TRUE" = "Rt value",

"occup_score" = "Occupational stressors",

"family_score" = "Family stressors",

"number_preexisting" = "Health stressors",

"symp_score" = "Symptom stressors",

"demo_score" = "Demographic stressors",

"exposure_score" = "Exposure stressors",

"sleep_score" = "Sleep stressors",

"feelingnot_well" = "Feeling unwell"

))

fit_stressed <- broom::tidy(geefit_stressed_ipw,conf.int = TRUE) %>% mutate(model = "Stressed") %>%

relabel_predictors(c(

"rt_greater_than_1TRUE" = "Rt value",

"occup_score" = "Occupational stressors",

"family_score" = "Family stressors",

"number_preexisting" = "Health stressors",

"symp_score" = "Symptom stressors",

"demo_score" = "Demographic stressors",

"exposure_score" = "Exposure stressors",

"sleep_score" = "Sleep stressors",

"feelingnot_well" = "Feeling unwell"

))

fit_anxious <- broom::tidy(geefit_anxious_ipw,conf.int = TRUE) %>% mutate(model = "Anxious") %>%

relabel_predictors(c(

"rt_greater_than_1TRUE" = "Rt value",

"occup_score" = "Occupational stressors",

"family_score" = "Family stressors",

"number_preexisting" = "Health stressors",

"symp_score" = "Symptom stressors",

"demo_score" = "Demographic stressors",

"exposure_score" = "Exposure stressors",

"sleep_score" = "Sleep stressors",

"feelingnot_well" = "Feeling unwell"

))

fit_tired <- broom::tidy(geefit_tired_ipw,conf.int = TRUE) %>% mutate(model = "Tired") %>%

relabel_predictors(c(

"rt_greater_than_1TRUE" = "Rt value",

"occup_score" = "Occupational stressors",

"family_score" = "Family stressors",

"number_preexisting" = "Health stressors",

"symp_score" = "Symptom stressors",

"demo_score" = "Demographic stressors",

"exposure_score" = "Exposure stressors",

"sleep_score" = "Sleep stressors",

"feelingnot_well" = "Feeling unwell"

))

stressor_ind <- c(1,7,4,9,5,6,8)

n_order <- c("Rt value",

"Occupational stressors",

"Family stressors",

"Health stressors",

"Symptom stressors",

"Demographic stressors",

"Exposure stressors",

"Sleep stressors",

"Feeling unwell"

)

fit_all<-rbind(fit_angry[stressor_ind,],fit_lonely[stressor_ind,], fit_sad[stressor_ind,],fit_stressed[stressor_ind,],fit_anxious[stressor_ind,],fit_tired[stressor_ind,])

fit_all$term <- factor(fit_all$term,levels = n_order[stressor_ind])

{dwplot(fit_all, vline = geom_vline(xintercept = 0, colour = "grey60", linetype = 2),dot_args = list(size = 2.5), whisker_args = list(size = 1)) +theme_bw()+theme(plot.title = element_text(face="bold"),text=element_text(size=17, face="bold"))}+scale_color_manual(values = c( "#00468BFF", "black", "#42B540FF", "#0099B4FF", "#925E9FFF", "#FDAF91FF"))

ggsave('/ncf/xlin_covid/Users/sshen/dat5_02_09/HWF_plots/stressor_neg.pdf',height = 6.5,width = 8)

save(fit_all,file = "/ncf/xlin_covid/Users/sshen/dat5_02_09/results/fit_stressor_neg.RData")

library(geepack)

library(dotwhisker)

library(dplyr)

library(ggsci)

library(RColorBrewer)

load('/ncf/xlin_covid/Users/sshen/dat5_02_09/reg_data1.RData')

dat_ipw_complete$income_cat <- relevel(dat_ipw_complete$income_cat, ref = "[0,4.49e+04)")

#happy, optimistic, hopeful, calm, grateful, thoughtful

#angry, lonely, sad, stressed, anxious, tired

geefit_happy_ipw<-geeglm(emotions_happy~occup_score + family_score + number_preexisting + symp_score + demo_score + exposure_score + rt_greater_than_1 + gender + before_5_am+after_5_am+regions+ month1+income_cat+ feeling+weekend +Case_rate +Death_rate+covid_tested+covid_positive+sleep_score , id=session_id, family=gaussian(), corstr="independence", data=dat_ipw_complete,weights=weights)

geefit_optimistic_ipw<-geeglm(emotions_optimistic~occup_score + family_score + number_preexisting + symp_score + demo_score + exposure_score + rt_greater_than_1 + gender + before_5_am+after_5_am+regions+ month1+income_cat+ feeling+weekend +Case_rate +Death_rate+covid_tested+covid_positive+sleep_score , id=session_id, family=gaussian(), corstr="independence", data=dat_ipw_complete,weights=weights)

geefit_hopeful_ipw<-geeglm(emotions_hopeful~occup_score + family_score + number_preexisting + symp_score + demo_score + exposure_score + rt_greater_than_1 + gender + before_5_am+after_5_am+regions+ month1+income_cat+ feeling+weekend +Case_rate +Death_rate+covid_tested+covid_positive+sleep_score , id=session_id, family=gaussian(), corstr="independence", data=dat_ipw_complete,weights=weights)

geefit_calm_ipw<-geeglm(emotions_calm~occup_score + family_score + number_preexisting + symp_score + demo_score + exposure_score + rt_greater_than_1 + gender + before_5_am+after_5_am+regions+ month1+income_cat+ feeling+weekend +Case_rate +Death_rate+covid_tested+covid_positive+sleep_score , id=session_id, family=gaussian(), corstr="independence", data=dat_ipw_complete,weights=weights)

geefit_grateful_ipw<-geeglm(emotions_grateful~occup_score + family_score + number_preexisting + symp_score + demo_score + exposure_score + rt_greater_than_1 + gender + before_5_am+after_5_am+regions+ month1+income_cat+ feeling+weekend +Case_rate +Death_rate+covid_tested+covid_positive+sleep_score , id=session_id, family=gaussian(), corstr="independence", data=dat_ipw_complete,weights=weights)

geefit_thoughtful_ipw<-geeglm(emotions_thoughtful~occup_score + family_score + number_preexisting + symp_score + demo_score + exposure_score + rt_greater_than_1 + gender + before_5_am+after_5_am+regions+ month1+income_cat+ feeling+weekend +Case_rate +Death_rate+covid_tested+covid_positive+sleep_score , id=session_id, family=gaussian(), corstr="independence", data=dat_ipw_complete,weights=weights)

fit_happy <- broom::tidy(geefit_happy_ipw,conf.int = TRUE) %>% mutate(model = "Happy") %>%

relabel_predictors(c(

"rt_greater_than_1TRUE" = "Rt value",

"occup_score" = "Occupational stressors",

"family_score" = "Family stressors",

"number_preexisting" = "Health stressors",

"symp_score" = "Symptom stressors",

"demo_score" = "Demographic stressors",

"exposure_score" = "Exposure stressors",

"sleep_score" = "Sleep stressors",

"feelingnot_well" = "Feeling unwell"

))

fit_optimistic <- broom::tidy(geefit_optimistic_ipw,conf.int = TRUE) %>% mutate(model = "Optimistic") %>%

relabel_predictors(c(

"rt_greater_than_1TRUE" = "Rt value",

"occup_score" = "Occupational stressors",

"family_score" = "Family stressors",

"number_preexisting" = "Health stressors",

"symp_score" = "Symptom stressors",

"demo_score" = "Demographic stressors",

"exposure_score" = "Exposure stressors",

"sleep_score" = "Sleep stressors",

"feelingnot_well" = "Feeling unwell"

))

fit_hopeful <- broom::tidy(geefit_hopeful_ipw,conf.int = TRUE) %>% mutate(model = "Hopeful") %>%

relabel_predictors(c(

"rt_greater_than_1TRUE" = "Rt value",

"occup_score" = "Occupational stressors",

"family_score" = "Family stressors",

"number_preexisting" = "Health stressors",

"symp_score" = "Symptom stressors",

"demo_score" = "Demographic stressors",

"exposure_score" = "Exposure stressors",

"sleep_score" = "Sleep stressors",

"feelingnot_well" = "Feeling unwell"

))

fit_calm <- broom::tidy(geefit_calm_ipw,conf.int = TRUE) %>% mutate(model = "Calm") %>%

relabel_predictors(c(

"rt_greater_than_1TRUE" = "Rt value",

"occup_score" = "Occupational stressors",

"family_score" = "Family stressors",

"number_preexisting" = "Health stressors",

"symp_score" = "Symptom stressors",

"demo_score" = "Demographic stressors",

"exposure_score" = "Exposure stressors",

"sleep_score" = "Sleep stressors",

"feelingnot_well" = "Feeling unwell"

))

fit_grateful <- broom::tidy(geefit_grateful_ipw,conf.int = TRUE) %>% mutate(model = "Grateful") %>%

relabel_predictors(c(

"rt_greater_than_1TRUE" = "Rt value",

"occup_score" = "Occupational stressors",

"family_score" = "Family stressors",

"number_preexisting" = "Health stressors",

"symp_score" = "Symptom stressors",

"demo_score" = "Demographic stressors",

"exposure_score" = "Exposure stressors",

"sleep_score" = "Sleep stressors",

"feelingnot_well" = "Feeling unwell"

))

fit_thoughtful <- broom::tidy(geefit_thoughtful_ipw,conf.int = TRUE) %>% mutate(model = "Thoughtful") %>%

relabel_predictors(c(

"rt_greater_than_1TRUE" = "Rt value",

"occup_score" = "Occupational stressors",

"family_score" = "Family stressors",

"number_preexisting" = "Health stressors",

"symp_score" = "Symptom stressors",

"demo_score" = "Demographic stressors",

"exposure_score" = "Exposure stressors",

"sleep_score" = "Sleep stressors",

"feelingnot_well" = "Feeling unwell"

))

stressor_ind <- c(1,7,4,9,5,6,8)

n_order <- c("Rt value",

"Occupational stressors",

"Family stressors",

"Health stressors",

"Symptom stressors",

"Demographic stressors",

"Exposure stressors",

"Sleep stressors",

"Feeling unwell"

)

fit_all <- rbind(fit_happy[stressor_ind,],fit_optimistic[stressor_ind,], fit_hopeful[stressor_ind,],fit_calm[stressor_ind,],fit_grateful[stressor_ind,],fit_thoughtful[stressor_ind,])

fit_all$term <- factor(fit_all$term,levels = n_order[stressor_ind])

{dwplot(fit_all, vline = geom_vline(xintercept = 0, colour = "grey60", linetype = 2),dot_args = list(size = 2.5), whisker_args = list(size = 1)) +theme_bw()+theme(plot.title = element_text(face="bold"), text=element_text(size=17, face="bold"))}+ scale_color_manual(values = c( "#00468BFF", "black", "#42B540FF", "#0099B4FF", "#925E9FFF", "#FDAF91FF"))

ggsave('/ncf/xlin_covid/Users/sshen/dat5_02_09/HWF_plots/stressor_pos.pdf',height = 6.5,width = 8)

save(fit_all,file = "/ncf/xlin_covid/Users/sshen/dat5_02_09/results/fit_stressor_pos.RData")
