# Supplementary material for: Daily positive and negative affect during the COVID-19 pandemic
Source: Front Psychol. 2024 Jan 8;14:1239123. doi: 10.3389/fpsyg.2023.1239123 (PMC10800618; doi:10.3389/fpsyg.2023.1239123)
Supplement: Supplementary file 2 [file Image_1.pdf]

Supplementary Figure 1

Associations between Affect and Dropout

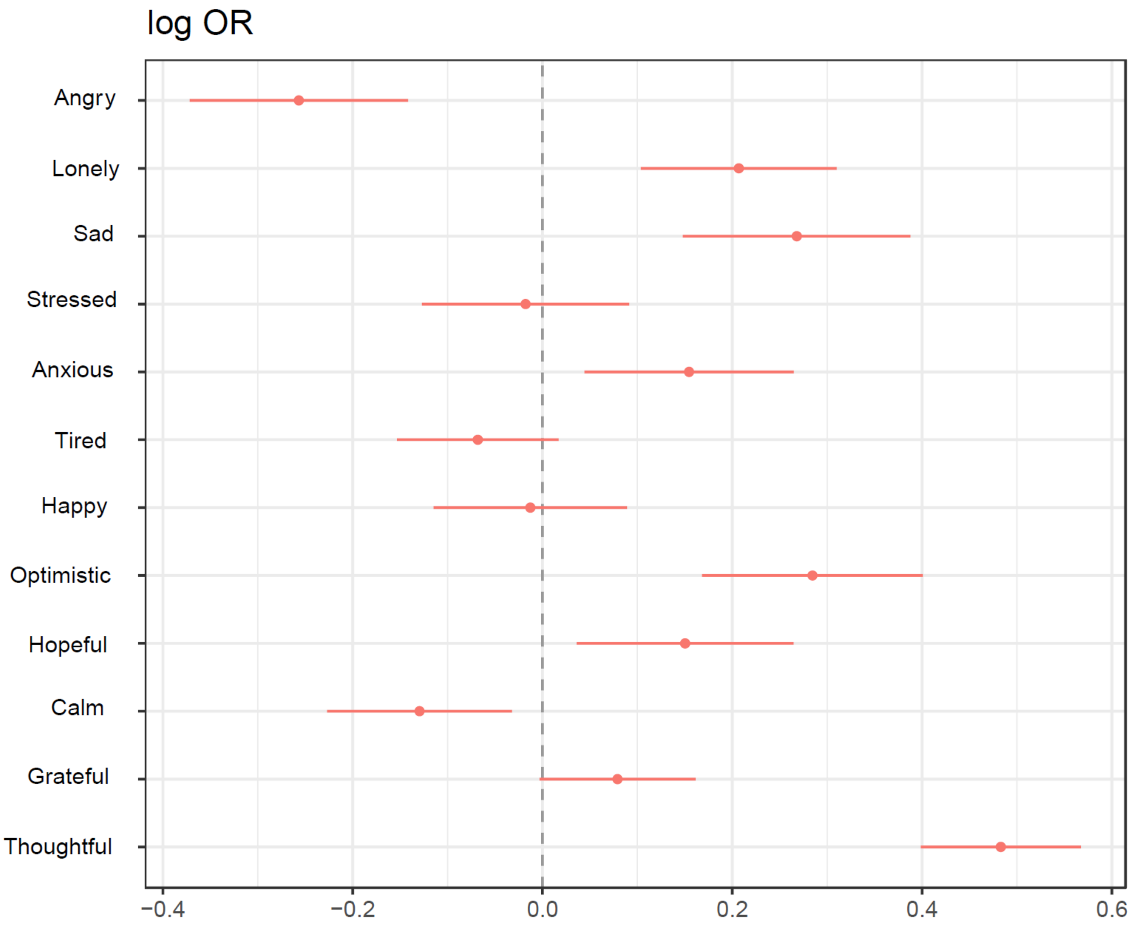

Note. A logistic regression was conducted to examine the associations between affect and dropouts (0 = dropout, 1 = continuing), adjusting for covariates (gender, ethnic groups, age, and months since joining the HWF). Shown are log odds ratios and the corresponding 95% confidence intervals.
