# Supplementary material for: Daily positive and negative affect during the COVID-19 pandemic
Source: Front Psychol. 2024 Jan 8;14:1239123. doi: 10.3389/fpsyg.2023.1239123 (PMC10800618; doi:10.3389/fpsyg.2023.1239123)
Supplement: Supplementary file 3 [file Image_2.pdf]

Supplementary Figure 2

Mean Positive (a) and Negative (b) Affect from May/June 2020 to February 2021.

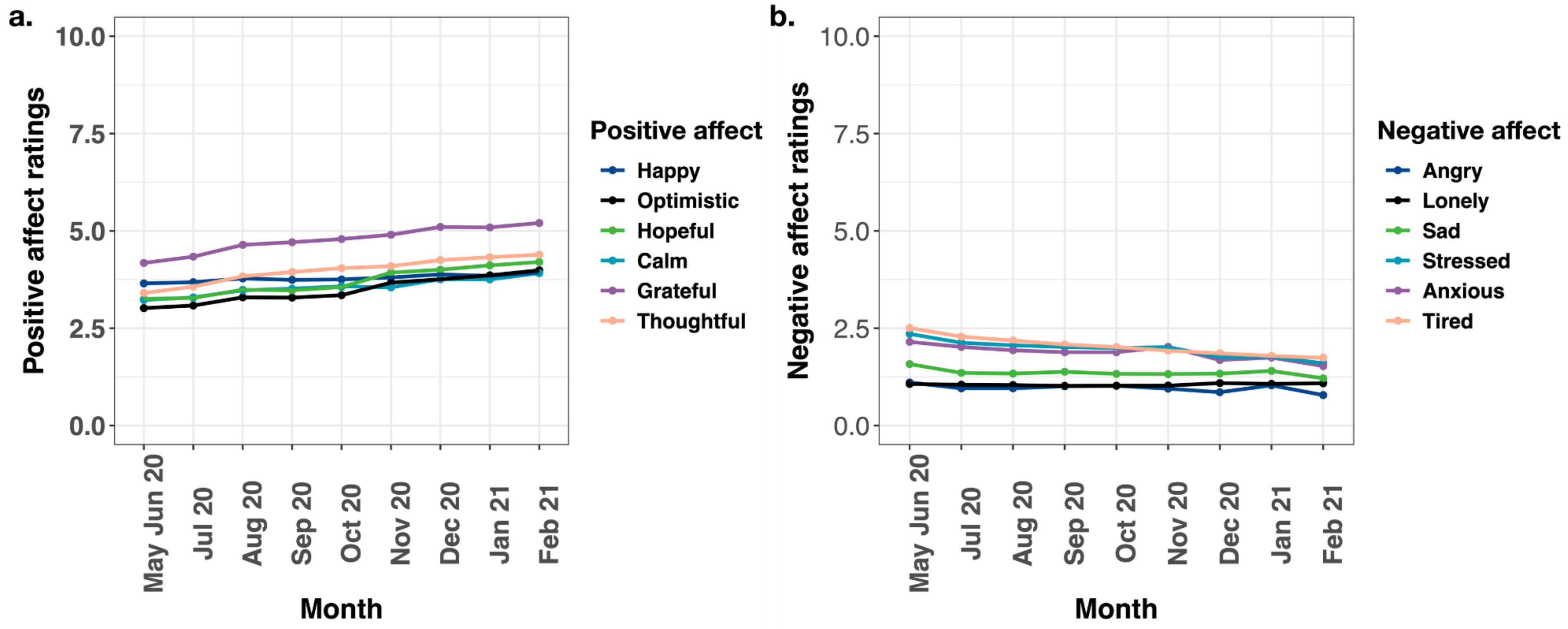

Note. The means were computed for all responses for each month (n = 3,509,982 responses from 151,049 users).
