# Supplementary material for: Daily positive and negative affect during the COVID-19 pandemic
Source: Front Psychol. 2024 Jan 8;14:1239123. doi: 10.3389/fpsyg.2023.1239123 (PMC10800618; doi:10.3389/fpsyg.2023.1239123)
Supplement: Supplementary file 4 [file Table_1.DOCX]

**Supplementary Table 1**

*Comparison of demographic groups in the U.S. and the study sample*

|  |  |  |  |
| --- | --- | --- | --- |
|  |  | U.S. Census 2020 | Study Sample |
| *Gender* | |  |  |
|  | Female | 50.40 | 80.00 |
|  | Male | 49.10 | 19.70 |
|  | other | not specified | 0.30 |
| *Race/ethnicity* |  |  |  |
|  | White | 75.50 | 79.80 |
|  | Black/African American | 13.60 | 4.00 |
|  | American Indian or Alaska Native | 1.30 | 0.40 |
|  | Asian | 6.30 | 2.80 |
|  | Native Hawaiian or Other Pacific Islander | 0.30 | 0.20 |
|  | Some other race | not specified | 0.80 |
|  | Two or more races | 3.00 | 5.10 |
| *Region* |  |  |  |
|  | Northeast | 17.30 | 33.00 |
|  | South | 38.10 | 25.00 |
|  | Midwest | 20.80 | 17.90 |
|  | West | 23.70 | 24.20 |
| *Household living* | |  |  |
|  | Living alone | 27.60 | 12.20 |

*Note*. Census data based on 2020 numbers: <https://www.census.gov/quickfacts/fact/table/US/PST045222>
