# Supplementary material for: Daily positive and negative affect during the COVID-19 pandemic
Source: Front Psychol. 2024 Jan 8;14:1239123. doi: 10.3389/fpsyg.2023.1239123 (PMC10800618; doi:10.3389/fpsyg.2023.1239123)
Supplement: Supplementary file 5 [file Table_2.DOCX]

Supplementary Table 2

*Sample Size in Each Month*

|  |  | |  | |  | |  | |  | |  |  | |  | |  | | |
| --- | --- | --- | --- | --- | --- | --- | --- | --- | --- | --- | --- | --- | --- | --- | --- | --- | --- | --- |
| Month | | May/Jun | | Jul | | Aug | | Sep | | Oct | | | Nov | | Dec | | Jan | Feb |
| May/June 2020 | | 134,700 | | 85,878 | | 61,852 | | 46,539 | | 37,786 | | | 32,311 | | 26,812 | | 23,380 | 15,351 |
| July 2020 | |  | | 11,386 | | 4,618 | | 3,153 | | 2,390 | | | 2,089 | | 1,684 | | 1,417 | 823 |
| August 2020 | |  | |  | | 4,237 | | 1,658 | | 1,153 | | | 911 | | 757 | | 622 | 357 |
| September 2020 | |  | |  | |  | | 334 | | 139 | | | 109 | | 87 | | 63 | 35 |
| October 2020 | |  | |  | |  | |  | | 140 | | | 63 | | 41 | | 27 | 19 |
| November 2020 | |  | |  | |  | |  | |  | | | 87 | | 40 | | 29 | 18 |
| December 2020 | |  | |  | |  | |  | |  | | |  | | 98 | | 50 | 20 |
| January 2021 | |  | |  | |  | |  | |  | | |  | |  | | 56 | 17 |
| February 2021 | |  | |  | |  | |  | |  | | |  | |  | |  | 11 |

Note. The table displays the number of users joined in each month from May/June 2020 to February 2023 and the number of the users who continued using the HWF app. For example, 134,700 joined in May/June and 85,878 of them continued in July.
